# Particle leakage through the exhalation valve on a face mask under flow conditions mimicking human breathing: A critical assessment

**DOI:** 10.1063/5.0067174

**Published:** 2021-10-25

**Authors:** Yeeun Kang, Jooyeon Park, Hyungmin Park

**Affiliations:** 1 Department of Mechanical Engineering, Seoul National University, Seoul 08826, Korea; 2 Institute of Advanced Machines and Design, Seoul National University, Seoul 08826, Korea

## Abstract

In today's era of active personal protections against airborne respiratory disease, general interest in the multiphase flow physics underlying face masks is greater than ever. The exhalation valves, installed on some masks to mitigate the breathing resistance, have also received more attention. However, the current certification protocol of evaluating airflow leakage only when suction pressure is applied is insufficient to capture practical aspects (particle penetration or leakage). Here, we experimentally measure two-phase flow across valve-type masks under conditions mimicking actual breathing. During exhalation, a high-speed jet through the valve accelerates the transmission of particles from inside while reasonable protection from external pollutants is achieved during inhalation, which supports the warnings from various public health officials. Based on the mechanism of particle penetration found here, we hope a novel design that both achieves high-efficiency shielding and facilitates easy breathing can be developed.

## I. INTRODUCTION

**FIG. 1.**
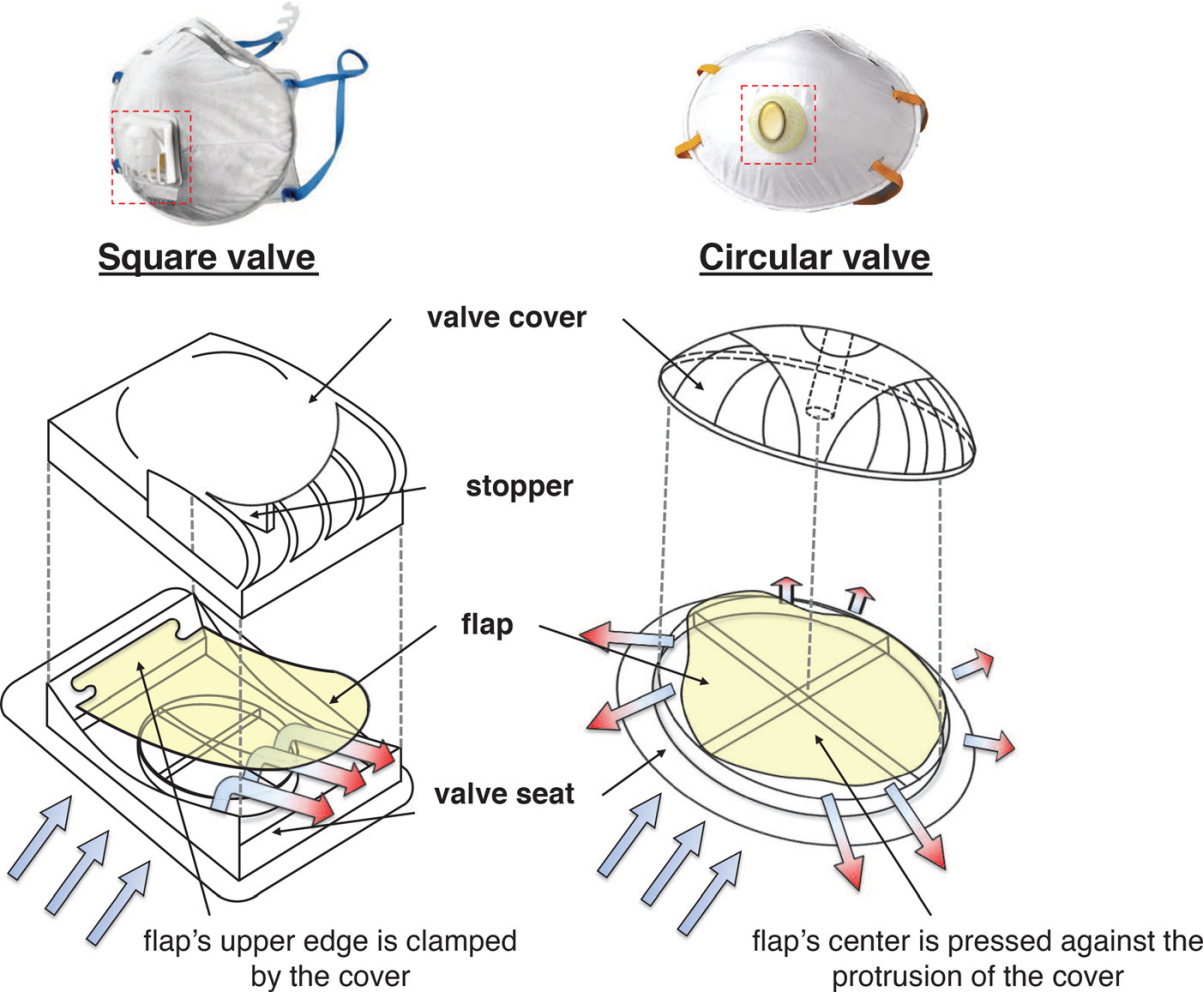
Illustration of the operating principle of exhalation valves depending on the valve type.

Except in some Asian countries with seasonal issue of particulate matters, face masks are generally used as personal protective gear in specialized environments such as medical institutions, hazardous work places, and cleanrooms. However, the importance of personal shielding against airborne respiratory disease during the global SARS-CoV-2 pandemic increased the public interest in face masks more than ever (Busco *et al.*, 2020; Cheng *et al.*, 2020; Dbouk and Drikakis, 2020a; Mittal *et al.*, 2020a, 2020b; Bourouiba, 2021; Mathai *et al.*, 2021). Scientific research (focusing on aspects of fluid mechanics) supporting and analyzing their functionality has flourished (Hui *et al.*, 2012; Arumuru *et al.*, 2020; Dbouk and Drikakis, 2020b; Pendar and Páscoa, 2020; Verma *et al.*, 2020a). Although many different types of face masks are available, some are equipped with an exhalation valve to facilitate breathing. As shown in Fig. 1, the exhalation valve is a unidirectional flow valve (Li and Liou, 2003; Nobili *et al.*, 2008; De Tullio *et al.*, 2009; Roberge, 2012) with a flap made of natural/silicone rubber or neoprene inside. As the user exhales, this flap opens outward, discharging humid air, resulting in a reduction in the pressure drop (drag) across the mask. In addition to the flap, the mask includes a valve seat and cover to hold the flap in place and to protect the device, respectively.

According to present regulations for the approval of the protective devices, established by the Korea Ministry of Employment and Laboratory, dust respirators with a filtration rate of 94% or higher should be equipped with an exhalation valve (common N95 respirators are equipped with an exhalation valve because of their higher filtration rate). Despite its importance to users' health, however, the current certification test employed by the National Institute for Occupational Safety and Health seems insufficient for rigorous evaluation of shielding performance under practical (i.e., realistic) conditions (Brosseau, 1998; Kuo *et al.*, 2005; Roberge *et al.*, 2012). According to 42 CFR part 84 (USA), air leakage into the respirator from the exhalation valve should not exceed 30 ml/min under a constant suction head of 25 mmH_2_O. Although the amount of air leakage is controlled, the aerodynamic behavior (concentration, dispersion, and trapping) of particles (solid, fluid, or biological; Tang *et al.,* 1992; Eaton and Fessler, 1994; Marchioli and Soldati, 2002; Gibert *et al.*, 2012; Liu *et al.*, 2020; Park and Park, 2021) that accompanies the operation of the valve in flow conditions mimicking actual human breathing is unknown. However, it is critical to ensure that the penetration of particles through the exhalation valve does not compromise the filtering efficiency of the face mask. Furthermore, a pressure drop as high as 25 mmH_2_O typically corresponds to a heavy workload (i.e., breathing flow rate ∼100 L/min; Hinds and Kraske, 1987; Chen *et al.*, 1993), and thus it is necessary to test in the range of wider (more common) pressure drop (Bellin and Hinds, 1990; Kuo *et al.*, 2005) or in a cyclic (unsteady) flow similar to that of actual human breathing (Brosseau, 1998).

Previous studies on particle leakage through the exhalation valve of a half-mask respirator have focused on the effect of deficiencies in the valve. Bellin and Hinds (1990) compared the penetration rate between normal and defected valves during inhalation by measuring the mass of leaked aerosol at various work rates of the wearer ranging from 0 to 622 kg·m/min. They showed that the penetration increased as the valve is more contaminated and the aerosol particle size decreases. A properly functioning exhalation valve permits the minimal inward leakage below 0.01%, but the penetration rate increased as high as 1.1% when the valve operation is hindered at the valve seat, and valves with paint or dust contamination showed a leakage of 0.2–0.6%. Kuo *et al.* (2005) considered a wider range of pressure drop ( 15–45 mmH_2_O) to measure the leakage through the exhalation valve on face mask, pointing out the limitation of conventional certification criterion. With four different types of exhalation valves, they investigated the leakage characteristics in relation to the pressure drop and also tested the effect of artificial defects of the valve—fiber insertion between the flap and valve seat and the dent on valve seat. For the valve with a normal condition, the leakage rate through the square-type valve increased with increasing the suction head, but that of the circular valve with a knob showed an opposite trend (see Fig. 1). However, the flow characteristics (and leakage of particles) of the normal (undamaged) exhalation valve have not been investigated in detail.

In the past, face masks were mainly worn to protect users against hazardous airborne substances, but nowadays the focus is on the source control together, which is essential to preventing the epidemics of respiratory infections (Hui *et al.*, 2012; Arumuru *et al.*, 2020; Cheng *et al.*, 2020). The U.S. Centers for Disease Control and Prevention recommends against wearing masks with exhalation valves or vents because they allow unfiltered exhaled air to escape, which increases the chance that respiratory droplets can be expelled to others. Verma *et al.* (2020b) performed qualitative visualizations to examine the performance of face shield and N95 mask equipped with an exhalation valve. When droplets of distilled water and glycerin (diameter is less than 10 μm) were expelled through mouth opening of manikin head with a valve mask, most of them were discharged through the valve deflected in the downward direction, eventually spreading into the surroundings. Staymates (2020) also visualized the flow through N95 respirator with and without an exhalation valve using the schlieren imaging and light scattering method. During exhalation, they observed a turbulent jet flow (exit velocity of 285 cm/s with the flow rate of 42 L/min) discharging through the valve in the downward direction. In addition, the N95 mask with a valve showed only 25–40% reduction of the discharged droplets compared to that of open pipe (no mask) case while regular N95 respirator without a valve achieved 95% reduction. All of these results support that the respirators with valve are not appropriate for the source control application.

Previous studies have been limited to statistical analyses of airflow leakage under constant suction (inhalation) or smoke visualization through the valve under constant ejection (exhalation). However because our ultimate aim is to develop a face mask that both achieves high-efficiency shielding and easy breathing, we need to understand how particles penetrate the valve beyond simple visualizations or statistical analyses. Knowledge of flow-induced particle dynamics, such as the preferential concentration and sweeping, is mature enough to tackle this issue (Leighton and Acrivos, 1987; Eaton and Fessler, 1994; Monchaux *et al.*, 2010; Lau and Nathan, 2014; Park and Park, 2021). Therefore, in this study, we experimentally investigate airflow through an exhalation valve and associated particle dynamics (penetration and concentration) mimicking realistic human breathing in terms of airflow rate and direction. Instead of just pushing a high-pressure air along the direction of inhalation, we test three separate cases: constant ejection (exhalation), constant suction (inhalation), and periodic ejection-suction (transition from exhalation to inhalation). In each case, the airflow rate is varied as *Q *=* *20, 50, and 80 L/min, representing normal breathing, moderate activity, and vigorous activity, respectively (Blackie *et al.*, 1991; Neder *et al.*, 2003; Janssen *et al.*, 2005; Gupta *et al.*, 2010). Airflow and particle concentration (penetration) were measured with adaptive particle image velocimetry (PIV) and planar nephelometry (PN), respectively. We pay attention to (i) particle dispersion through the valve in the ejection case (i.e., the transmission of infectious particles from inside) and (ii) penetration of particles from the outside in the periodic (pulsating) flow condition. A recent numerical study showed that particle dynamics such as penetration and dispersion can be enhanced by secondary flow caused by the pulsating nature of breathing (Monroe *et al.*, 2021). In terms of valve geometry, we considered the most representative types of valves—square and circular—among those commercially available. We believe that elucidating particle behavior induced by breathing flow around the exhalation valve will enable the development of a novel mask that achieves high-efficiency protection against airborne respiratory disease and facilitates breathing.

This paper is organized as follows. In Sec. II, we explain the experimental setup, including the measurement of airflow and particle concentration. We also provide details about how we calculate the particle penetration rate. In Sec. III, we discuss airflow and particle behavior focusing on particle-jet interaction and penetration patterns in each flow condition (constant ejection, suction, and transition flow). We further discuss the effect of the valve cover and estimate flow rates through mask filter and valve in Sec. IV. Finally, a summary and suggestions for an improved valve design are given in Sec. V.

## II. EXPERIMENTAL SETUP AND PROCEDURE

### A. Setup of the flow test facility

**FIG. 2.**
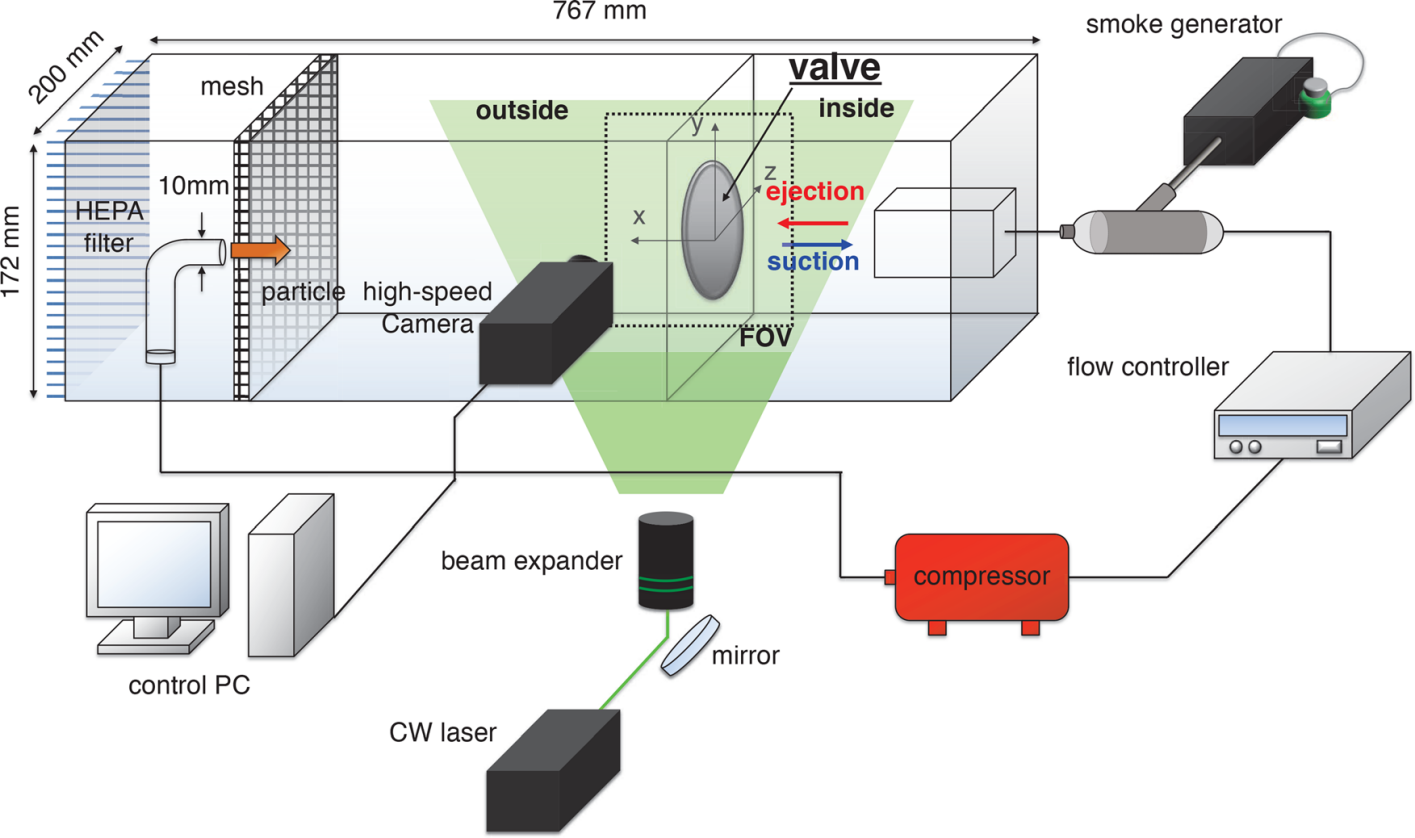
Experimental setup for the measurement of airflow and particle concentration with adaptive particle image velocimetry and high-speed imaging.

**FIG. 3.**
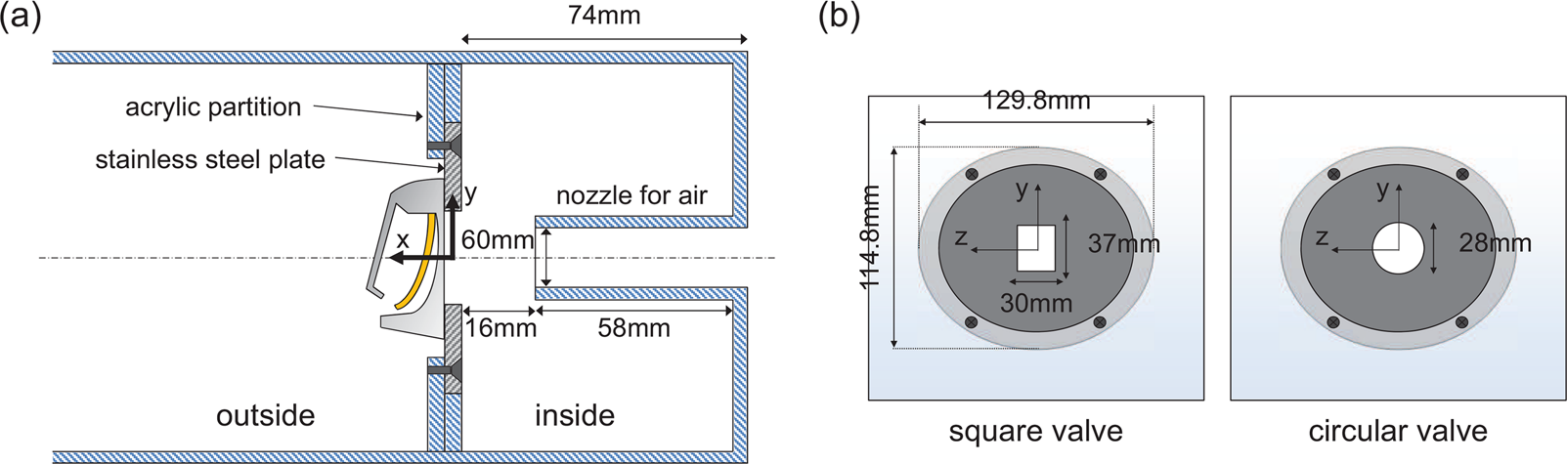
(a) Configuration of the valve and nozzle installed in the test chamber. (b) Details of the valve installation.

Figure 2 shows the experimental setup for measuring particle-laden flow across the exhalation valve while varying the airflow. Experiments are conducted in a 5 mm-thick transparent acrylic chamber, 767×172×200 mm^3^ in the streamwise (*x*), vertical (*y*), and transverse (*z*) directions, respectively. Inside the chamber, a partition wall with a hole for the valve installation separates the inside and outside of the valve (mask). On the inner side of the valve, a rectangular nozzle (70 mm wide and 60 mm high) is located in line with the opening for valve (typically, the exhalation valve is aligned with the oral cavity; Roberge *et al.*, 2010) [Fig. 3(a)]. The jet nozzle exit was located 16 mm from the valve, similar to the typical distance between the mouth and the valve ( 15–25 mm) in actual use. For the valve attachment, stainless steel plates with holes were overlapped and fixed to the acrylic partition to withstand the negative pressure generated inside by the suction (Fig. 3). Bi-directional jet flow, controlled by two solenoid valves, was designed to represent a combined nasal and oral breathing. We simulate human breathing inside the N95 respirator by applying the airflow in a confined space inside the partition wall in a range of Q=20–80 L/min in three different conditions: constant ejection (exhalation), constant suction (inhalation), and periodic ejection-suction (transition from exhalation to inhalation). A circular nozzle with a diameter of 10 mm was installed opposite the valve to inject particles into the chamber. It is noted that our experimental configuration was intended to represent a combined nasal and oral breathing, not just the flow from the nostril. As it is known that mouth opening area is approximately 1.7–2.1 times wider than nose opening area (Gupta *et al.*, 2010), we installed the pipe in line with the exhalation valve considering the position of the oral cavity (Roberge *et al.*, 2010). Previous studies on human breathing flows also modeled the oral breath through a pipe without an specific angle (Staymates, 2020; Verma *et al.*, 2020b). Also, a high-efficiency particulate air filter was attached on the end wall of the testbed to filter out any particles used in the measurements. This filter ensures a sufficiently small pressure drop ( 249.1–498.2 Pa) to almost mimic the conditions of the outside atmospheric.

For the valve, we used two representative models among those commercially available (square and circular valves, see Fig. 1). The valves were isolated from real masks with their covers removed and installed on the partition wall (Figs. 2 and 3). This ensures clear observation of the flapping motion of the valve and the associated particle-laden flow. As is discussed later in Sec. IV, the presence of a cover does not affect the present results. For the square valve, the upper edge of the flap is clamped by the valve cover, and a triangular structure protruding from the cover (called a stopper) holds the flap from opening beyond a certain degree. For the circular (or mushroom-type) valve, the round flap is held in place as its central part pressed against the protrusion of the cover (Fig. 1). The hydraulic diameter (*D*) of the flap for both types is 30 mm. To guarantee the same valve operation as in the normal environment, even after removing the valve cover, we confirm that the operation (i.e., movement) of the valves is not affected. For example, for the square valve, the upper edge of the flap was glued to the valve seat and stopper was installed to prevent excessive opening of the flap. For the circular valve, the equivalent portion of the flap fixed by the cover was glued to the seat with an epoxy.

**FIG. 4.**
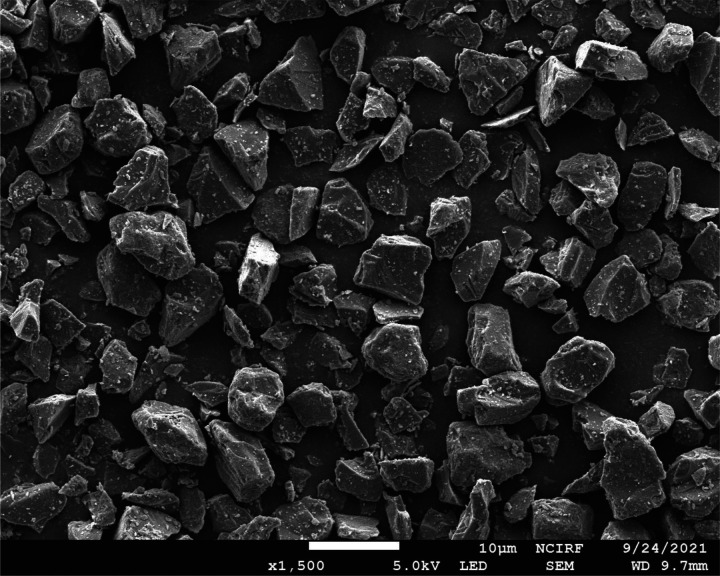
SEM image ( ×1500) of the silicon particles considered in the present study.

We used silicon particles (density $\rho_{p}=2.33$ g/cm^3^) with a nominal diameter of d=6 μm and a resistivity of 2140  Ω·m, and the contact angle of the water droplet is 87.5° (Bryk *et al.*, 2020). Figure 4 shows the SEM (scanning electron microscopy) images of the considered particles. The particles we used are expected to interact with the background flow in a manner similar to fine dust pollutants, bio-aerosol (virus), or small respiratory droplets, as judged by Stokes number ( $St=\tau_{p}/\tau_{f}\ll 1.0$), where *τ_p_* and *τ_f_* are the particle response time and flow timescale, respectively. Depending on the Stokes number, the particle behaviors in relation to the carrier fluid flow are characterized; when St≪1.0, particles behave almost like the flow tracer that faithfully follow the background flow, and with St≫1.0, the suspended particles react slowly or irrelevant to the surrounding flow. Thus, in general, micro-sized particles remain trapped in the vortical structures while larger ones are ejected out of them (Elgobashi, 2006). Here, the particle response time was calculated as $\tau_{p}=\rho_{p}d^{2}/18\rho_{f}\nu$, which is valid for very small particles of which the particle Reynolds number is less than 1.0 (Schiller and Neumann, 1933). *τ_p_* is derived based on the force balance between the Stokes drag force and gravitational body force on the particle under the condition of $\rho_{p}\gg \rho_{f}$. The flow timescale was defined as $\tau_{f}=D_{h}/U$, where *D_h_* is the hydraulic diameter of the jet nozzle and *U* is the jet bulk velocity (Fessler and Eaton, 1997; Lau and Nathan, 2014; Park and Park, 2021). In previous studies, silicon particles were used to mimic airborne contaminants: dusts (PM_2.5_), fumes, mist (solution being sprayed), microplastics, and bioaerosols (small respiratory droplets, and viral particles) (Bakand *et al.*, 2005; Yang *et al.*, 2007; Morawska *et al.*, 2009; Johnson *et al.*, 2011; Prata, 2018). Droplets less than 10 μm evaporate rapidly (in 0.1–1.0 s) at room temperature, form nuclei, and are transported as passive scalars (Bhagat *et al.*, 2020; Jarvis, 2020; Mittal *et al.*, 2020b). The particle size is evaluated with a particle size analyzer (Mastersizer 2000; Marvern Panalytical Ltd.) for a sample (10 g) of particles, and it has a Gaussian distribution with a standard deviation of 15% (Park and Park, 2021). In Table I, we provide important parameters characterizing the present experimental and flow conditions. Depending on the condition, the solid particles were loaded differently: for the ejection and suction/periodic cases, they were introduced upstream of the ejection square jet and outside the valve, respectively. Measurements were conducted after the particles are distributed uniformly. For each trial, we added 0.2 g (±0.02) of particles, corresponding to the one-way coupling regime ( $\Phi \leq 10^{-6}$), where the particle volume fraction Φ is the ratio of the total volume of solid particles to that of the test section; thus, we can ignore the modulation of continuous-phase flow by the particles (Elgobashi, 2006).

**TABLE I.** Summary of the considered experimental parameters and dimensionless numbers. Here, the Reynolds number is defined as $Re_{D}=UD/\nu$ (*D*: valve height; *ν*: air kinematic viscosity; *U*: jet bulk velocity).

Case	*Q* (L/min)	*U* (m/s)	*Re_D_*	*St*	Breathing condition
Ejection	20	0.0794	160	$3.18\times 10^{-4}$	Normal breathing
	50	0.1984	400	$7.95\times 10^{-4}$	Moderate activity
	80	0.3175	630	$1.27\times 10^{-3}$	Vigorous activity
Suction	20	0.0794	160	$3.18\times 10^{-4}$	Normal breathing
	50	0.1984	400	$7.95\times 10^{-4}$	Moderate activity
	80	0.3175	630	$1.27\times 10^{-3}$	Vigorous activity
Periodic	30	0.1190	240	$4.77\times 10^{-4}$	Normal breathing
	50	0.1984	400	$7.95\times 10^{-4}$	Moderate activity
	80	0.3175	630	$1.27\times 10^{-3}$	Vigorous activity

### B. Measurement of airflow velocity

To measure airflow across the valve, we used particle image velocimetry (PIV) (Fig. 2). For the tracers, high-purity liquid polyol (Fog Fluid Standard; Dantec Dynamics) is atomized into oil droplets (nominal diameter ∼1 μm) with a smoke generator (Safex; Dantec Dynamics) and fed into the airflow. A 5 W continuous-wave laser (RayPower 5000; Dantec Dynamics) with a wavelength of 532 nm was used as a light source to illuminate the tracers on the measurement plane. A high-speed camera (SpeedSense M310; Dantec Dynamics) equipped with a 100 mm lens (Nikon) was used to capture the tracers, at a rate of 6000 fps with a resolution of 640 × 800 pixels. Since the valve shape is symmetric against the z−axis, the flow measurements were made on the center (*z *=* *0) plane. The field of view (FOV) has a range of −0.6≤x/D≤0.6 and −0.7≤y/D≤0.7 with the center of the valve as the origin. The spatial resolution of the measurement is 0.00186D. To reliably cover the wide range of velocity magnitudes in the FOV, we used adaptive particle image velocimetry (Dynamic studio; Dantec Dynamics). This algorithm iteratively adjusts the size, shape, and location of individual interrogation windows (IWs) to compensate for locally deviating densities of seeding particles, flow velocities, and velocity gradients by setting the minimum and maximum IW size.

Typically, the flow velocity (*u*) obtained from PIV measurement can be expressed as u=MΔs/Δt, where *M* denotes the magnification factor, Δt is the time interval between consecutive images evaluated, and Δs is the particle displacement during Δt. To estimate uncertainty in *u*, the contribution of each factor needs to be considered comprehensively. According to error propagation analysis (Clifford, 1973), uncertainty in velocity measurement is obtained as $\delta (u)=\sqrt{\delta {\left(M\right)}^{2}+\delta {\left(\Delta s\right)}^{2}+\delta {\left(\Delta t\right)}^{2}}$. Here δ(α) denotes the percent error in obtaining *α* (Lawson *et al.*, 1999; Kim *et al.*, 2015; Choi and Park, 2018). In the present setup, δ(M) is 0.13% with M=55.84 μm/pixel. For the time interval, the error was calculated as 0.3% with an inter-frame time interval of 0.5 μs for the high-speed camera and an actual time interval between the image pair of 0.167 ms (corresponding to 6000 fps). Finally, pixel resolution, universally affecting the particle displacement, is known as 0.1 pixel, and the percentage error for particle displacement appears as 3.22% through a comparison with average particle displacement (Keane and Adrian, 1990; Kim *et al.*, 2015; Kim and Park, 2019; Maeng and Park, 2021). Thus, the overall uncertainty was estimated as 3.24% for the present velocity measurement. However, the solid particles (not tracers) used to measure particle penetration and concentration have the Stokes number; $St=3.18\times 10^{-4}–1.27\times 10^{-3}$, which is sufficiently low to ascertain that particle dynamics will be closely determined by interactions with the airflow (Balachandar and Eaton, 2010). This characteristic particle dynamic is also valid for most known hazardous airborne particulate matters, including biological ones (Bakand *et al.*, 2005; Yang *et al.*, 2007; Morawska *et al.*, 2009; Prata, 2018; Bhagat *et al.*, 2020).

### C. Measurement of particle penetration and concentration

**FIG. 5.**
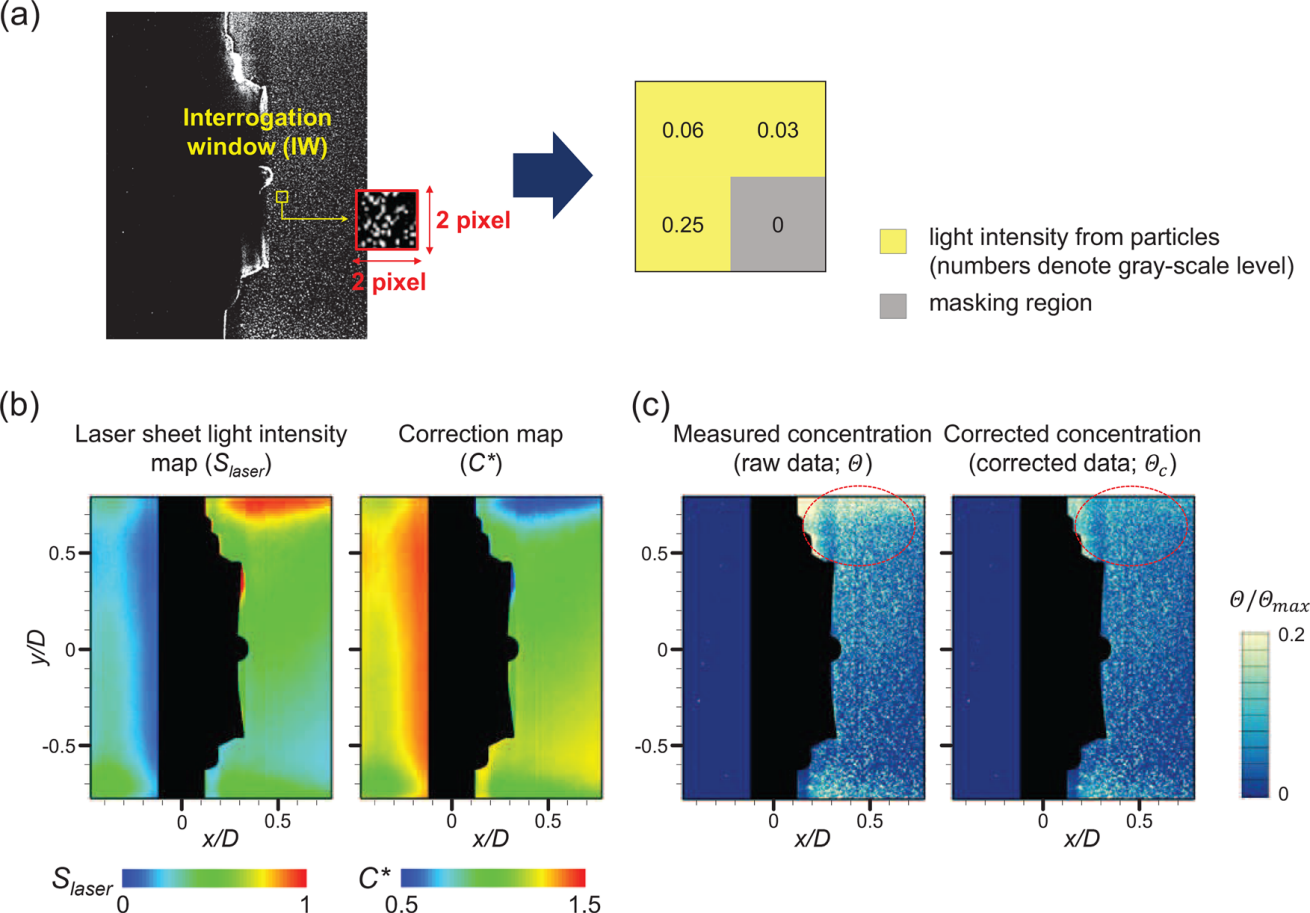
Particle concentration measured by planar nephelometry (PN): (a) raw image of dispersed particles in circular valve and a schematic diagram to calculate light intensity of interrogation window; (b) correction of non-uniform laser light sheet; and (c) correction of measured particle concentration.

In the same region of interest (ROI) for the airflow measurement, the particle distributions were measured using planar nephelometry (PN) (Birzer *et al.*, 2012; Lau and Nathan, 2014; Park and Park, 2021) (Fig. 5). We used silicon particles (size of 6 μm) comparable to airborne particulate matters (fine dust, fumes, and microplastics) and bioaerosols (small respiratory droplets, and viral particles) and the small Stokes number of $O(10^{-4})-O(10^{-3})$ indicates that the particle dynamics are mostly aerodynamic (Bakand *et al.*, 2005; Yang *et al.*, 2007; Morawska *et al.*, 2009; Prata, 2018; Bhagat *et al.*, 2020). The basic idea behind this method is to measure the distribution of light intensity that is proportional to particle number density, owing to the Mie scattering signal of the incident laser sheet, reflected from the particles. Thus, it is possible to quantify the particle concentration from the gray-level value of each pixel of the raw image. By defining an interrogation window (IW) of 2 × 2 pixels (50% overlap), we obtained the particle concentration (Θ) of each IW with Θ(x,y)= (sum of the gray-level values contained in IW)/(IW area), as shown in Fig. 5(a). Note that the regions occupied by the valve (including the moving flap) were masked before processing. The size of the IW was selected to avoid missing even a small number of particles that pass through the valve.

Since the laser sheet generally has a spatially non-uniform power intensity, it may distort the particle concentration measured based on the relative light intensity (Kalt and Nathan, 2007). Thus, we corrected for the effect of a biased laser power intensity distribution on the particle concentration measurement, using the method established by Park and Park (2021). As shown in Fig. 5(b), we measured the light intensity (expressed as the gray-level of each pixel) distribution of the applied laser sheet in the FOV by introducing oil droplets uniformly into the chamber. Then the time-averaged laser power intensity profile (*S_laser_*) is calculated, which is non-dimensionalized by its own maximum value in the FOV. Based on this non-uniform distribution of $S_{\mathrm{laser}}(x,y)$, the correction factor ( $C^{*}$) can be defined for each IW, as $C^{*}(x,y)=1-S_{\mathrm{laser}}(x,y)+S_{m}$, where $S_{m}\;(=0.5)$ is the median value of *S_laser_*. Finally, the concentration field data for each IW are corrected by $\Theta_{c}(x,y)=\Theta (x,y)\cdot C^{*}(x,y)$. The corrected concentration is further normalized as $\Theta_{c}/\Theta_{c,\max}$, by its maximum concentration value in the FOV, for a clearer illustration of the particle distribution; thus, the instantaneous concentration field level ranges in 0–1.0. Note that this correction is intended to prevent any optical distortion attributed to not only the biased laser intensity distribution but also the light reflection from objects in the setup [see the areas highlighted with a dashed circle in Fig. 5(c)].

### D. Calculation of the particle penetration rate

To quantify the amount of particle penetration (leakage) through the valve, we defined it based on the particle concentration field (Θ_*c*_); that is, temporal variation in the rate of penetration inside (*ψ_i_*) and outside (*ψ_o_*) the valve was calculated separately. Penetration inside the mask valve, *ψ_i_*, is defined as $\Sigma_{\mathrm{inside}}\Theta_{c}(t)/\Sigma \Theta_{c,\mathrm{initial}}$ to represent the number of particles penetrating through the valve present in the FOV. In the same way, particle penetration outward from the valve, *ψ_o_*, is calculated as $\Sigma_{\mathrm{outside}}\Theta_{c}(t)/\Sigma \Theta_{c,\mathrm{initial}}$ to indicate the number of particles present outside the valve in the FOV. The values of each penetration rate were further normalized by the concentration of particles in the FOV at the initial instant of measurement ( $\Sigma \Theta_{c,\mathrm{initial}}$) such that the sum of *ψ_i_* and *ψ_o_* is 1.0. The timescale (*t*) of the evaluation was expressed in real timescale (s).

## III. AIRFLOW AND PARTICLE BEHAVIOR UNDER FLOW CONDITIONS SIMULATING HUMAN BREATHING

### A. Constant ejection (exhalation)

**FIG. 6.**
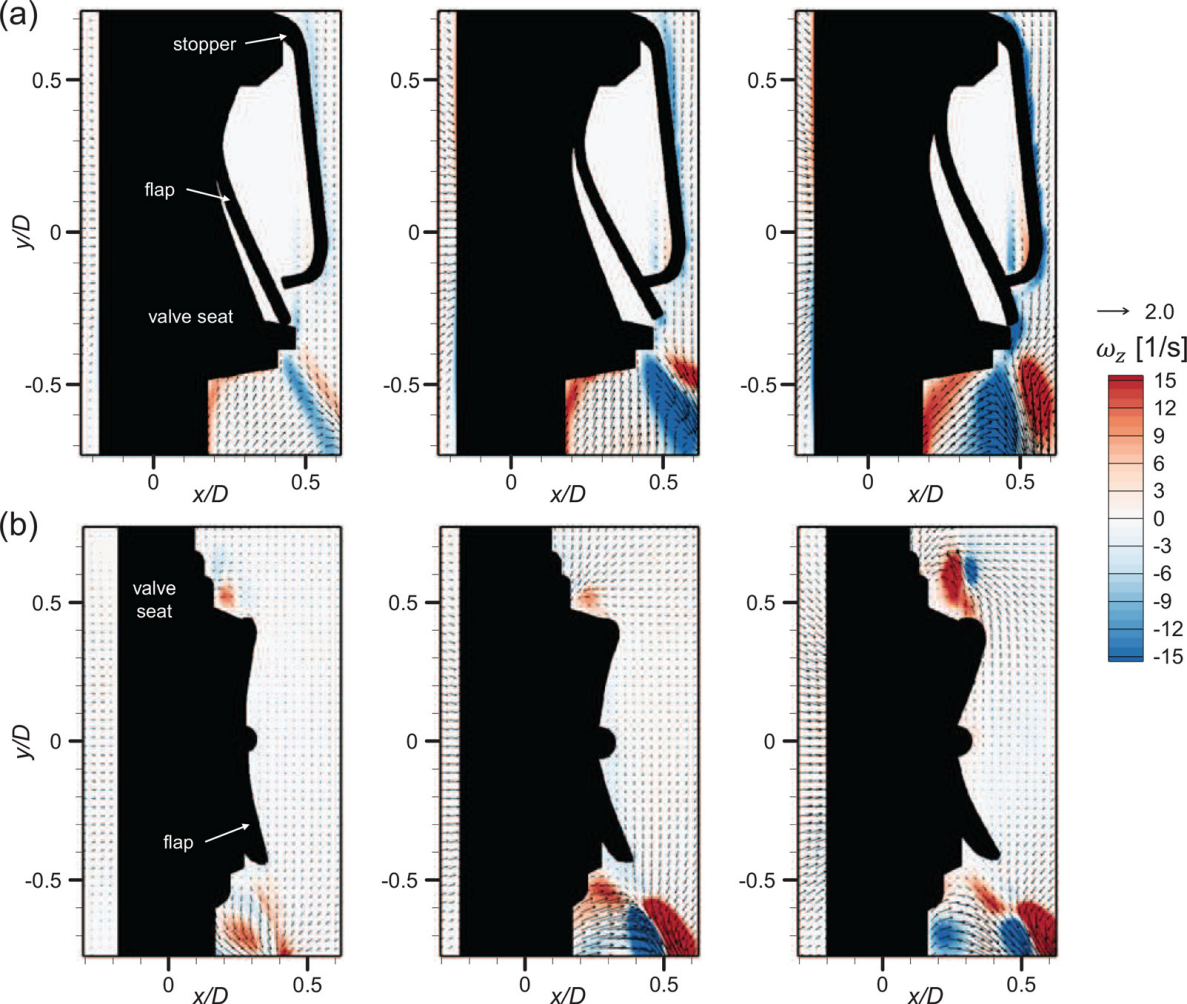
Velocity vectors and vorticity contour in the time-averaged airflow in ejection condition. Each corresponds to *Q *=* *20, 50, and 80 L/min from the left: (a) square valve and (b) circular valve.

First, we discuss the exhalation situation, which is represented by the constant ejection airflow. The most important feature in terms of the flow dynamics across the valve (or mask) under constant ejection is the formation of a high-speed jet through the narrow opening, which was mentioned by Verma *et al.* (2020b) and Staymates (2020). According to our measurements, the sudden decrease in the flow passage area (the gap between the flap and the valve seat) causes the ejection flow ( ∼0.08–0.3 m/s at the nozzle exit, which is similar to human breathing) to evolve into a highly accelerated (more than 20 times) jet ( ∼1.75–6.04 m/s at the valve exit). Previously, Staymates (2020) measured a flow velocity of 2.85 m/s (for an airflow rate of 42 L/min) from N95 respirator equipped with a square valve. Figure 6 shows time-averaged airflow fields with velocity vectors and vorticity contour according to the airflow rate *Q*. Since such a high-speed jet flow is generated along the direction of the flap opening, it appears differently depending on the valve type. For the square valve [Fig. 6(a)], in which the upper edge of the flap is clamped, the ejected air instantly lifts the lower part of the flap from the valve seat, generating a high-speed jet along the downward direction. When the airflow rate increases ( Q≥50 L/min), the lifted flap touches the rigid stopper (which restricts the flap from opening excessively) and is pressed slightly inward. However, for the circular valve, in which the central part of the flap is fixed, the gap between the flap and valve seat is created globally along the circumference of the flap [Fig. 6(b)]. For both valve types, a considerable flux of air is entrained into the jet from the outside, and this kind of flow field appears consistently while the airflow rate varies. As the airflow rate increases, the gap by the flap opening grows larger inducing a faster jet flow.

**FIG. 7.**
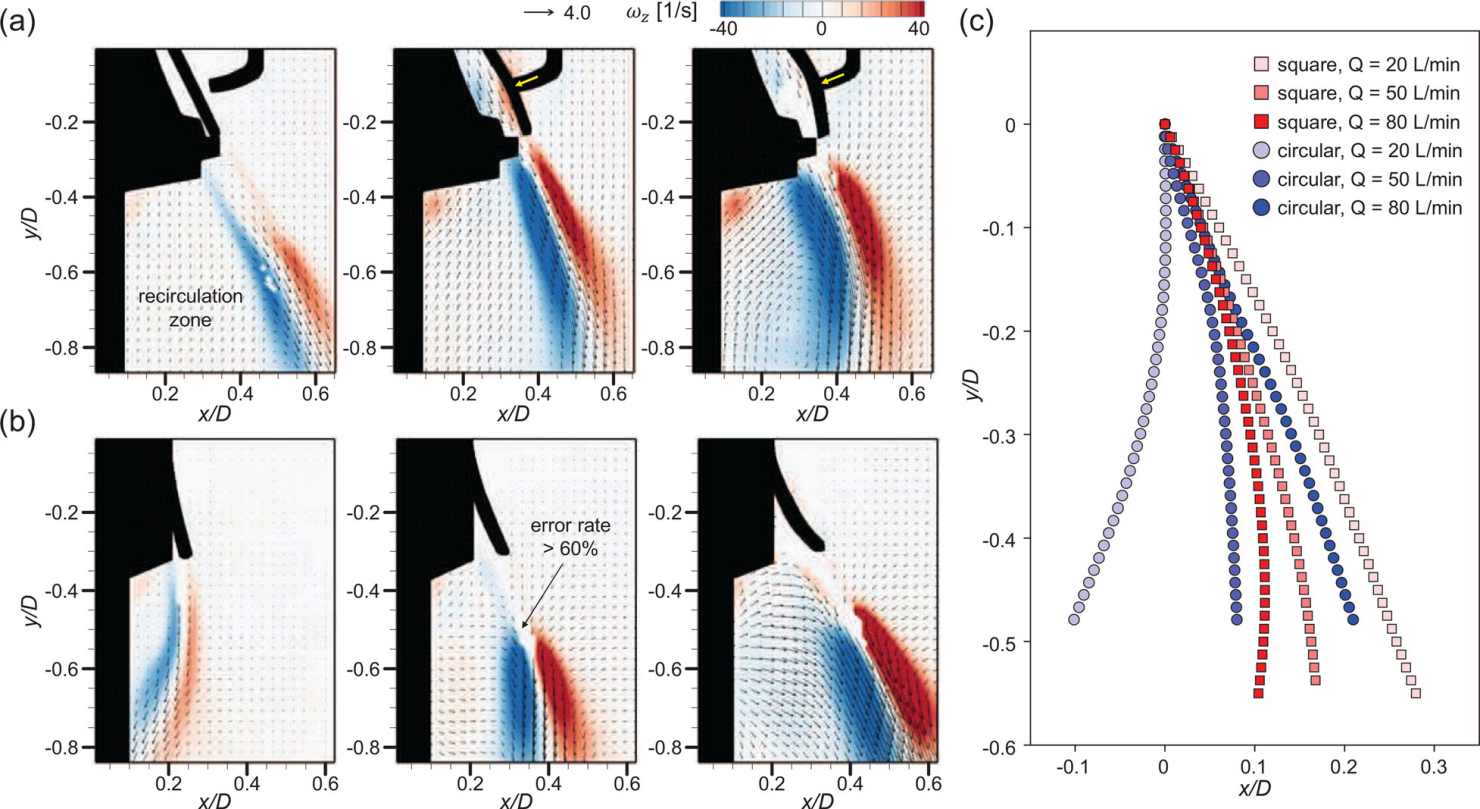
Velocity vectors and vorticity contour of the time-averaged high-speed jet flow in the ejection condition: (a) square valve and (b) circular valve. Vectors with an error rate over 60% have been excluded. Each sub-figure corresponds to *Q *=* *20, 50, and 80 L/min from the left. (c) Trajectory of the high-speed jet centerline.

**FIG. 8.**
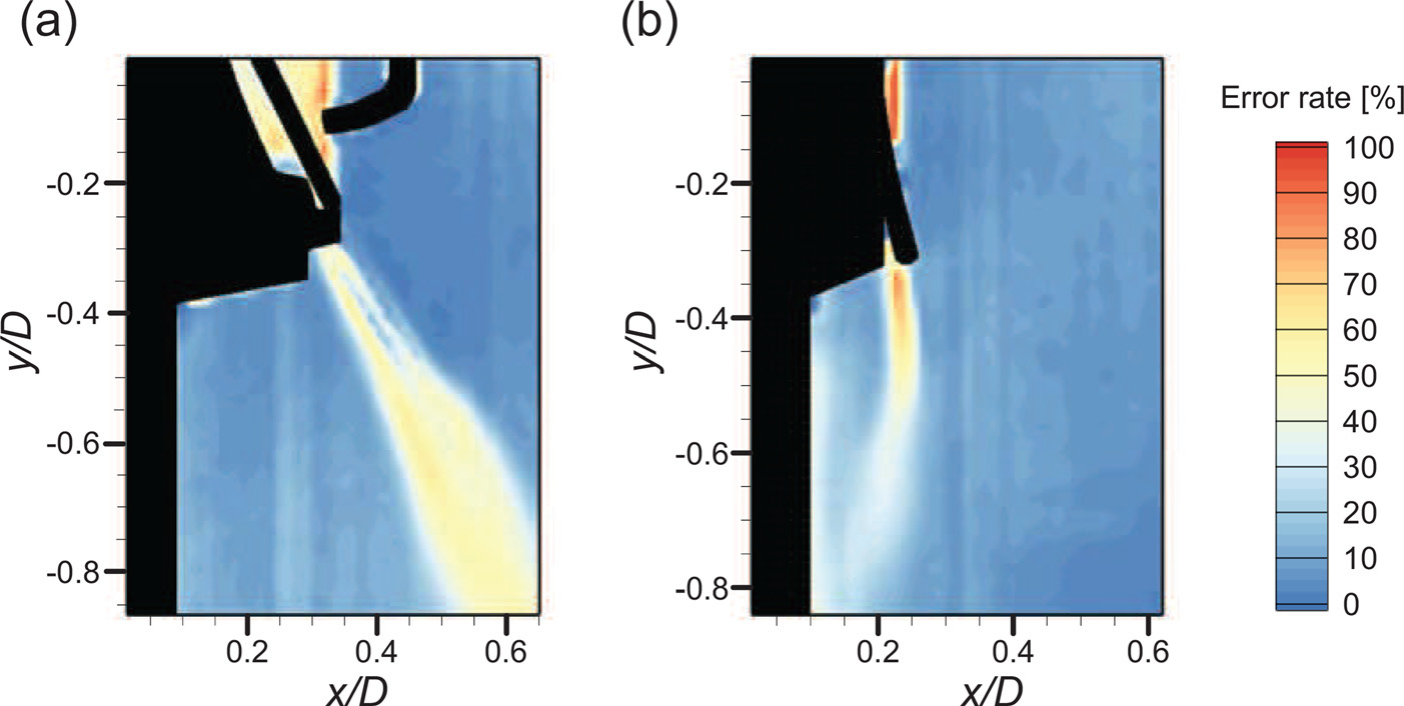
Error rate contour for the high-speed jet velocity field (*Q *=* *20 L/min): (a) square valve and (b) circular valve.

To observe the detailed flow structures involved with the high-speed jet, we additionally measured the flow velocity near the gap, as shown in Figs. 7(a) and 7(b). For both the square and circular valves, the jet shear-layer vortices show up as a counter-rotating vortex pair along the high-speed jet. Note that velocity vectors with an error rate greater than 60% are excluded (masked) from the time-averaged field. These error vectors are caused by the limited time resolution of the present velocity measurement method and mostly locates near the gap exit. Here, we calculate the error rate as the ratio of outliers identified from each instantaneous velocity field (Westerweel and Scarano, 2005) while obtaining the time-averaged flow fields (see Fig. 8). Figure 7(c) shows the trajectories of the jet centerline with Q, which was determined by the streamline starting from the jet exit, i.e., the location of the flap opening in the present configuration (Yuan and Street, 1998; Su and Mungal, 2004). It is interesting that the evolution of the jet from each valve type is directed differently with increasing *Q*. For the square valve, the jet is deflected inward as the airflow becomes stronger, since the flap is pressed harder by the stopper and the lower part is rolled inward. In contrast, the jet from the circular valve extends outward with increasing *Q*. The jet centerline velocity is in general higher for the circular valve because the gap is wider for the square valve while it is opened locally, which indicates that the faster jet is generated through the circular exhalation valve than the square valve under the same *Q*.

**FIG. 9.**
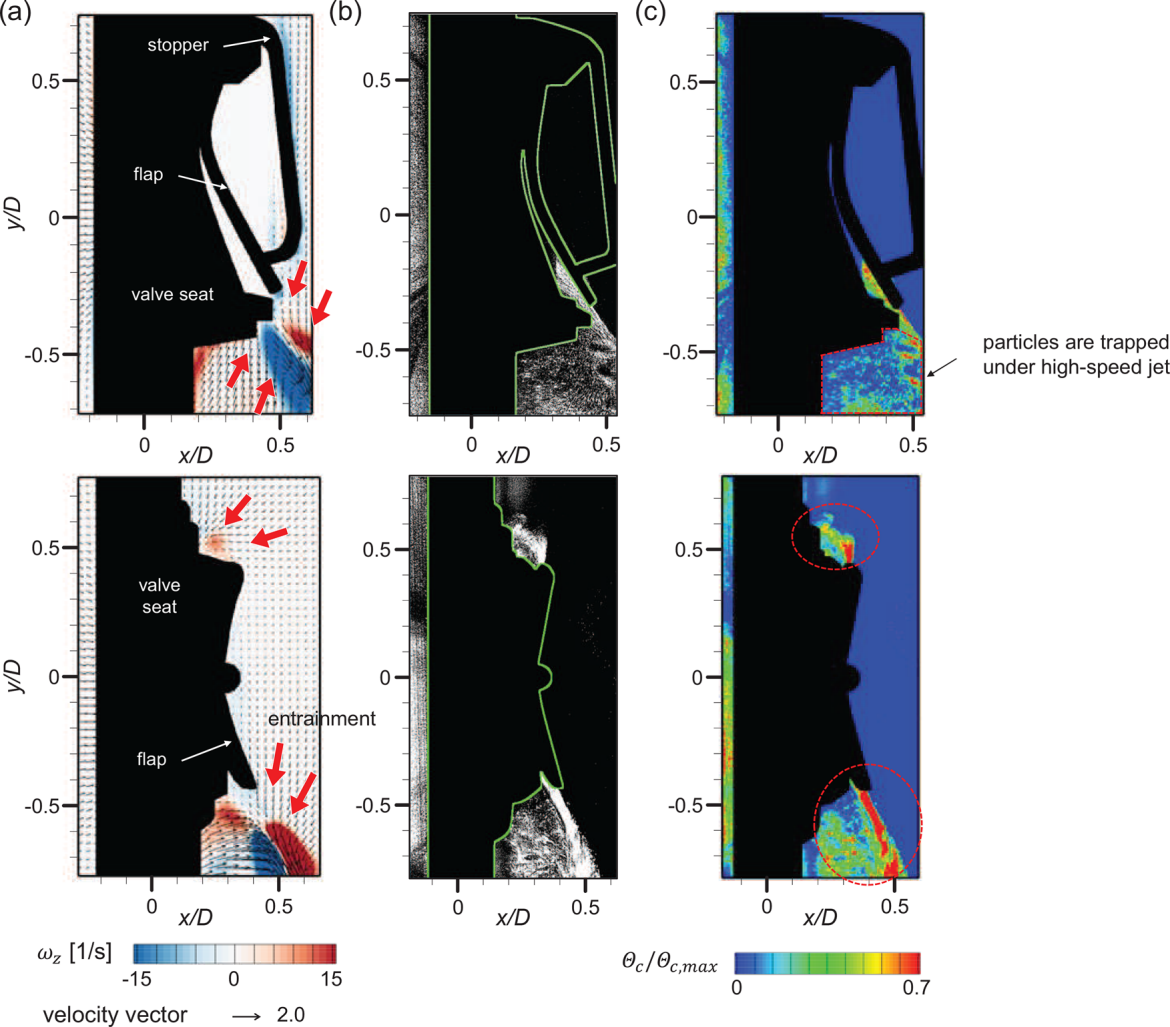
Particle dispersion mechanism in the ejection state (*Q *=* *50 L/min) for the square (top) and circular (bottom) valves: (a) time-averaged airflow velocity field; (b) instantaneous particle distribution (raw image); and (c) instantaneous particle distribution (concentration field).

The particle dispersion (or spreading) pattern through the valve under the exhalation condition is shown in Fig. 9 in terms of the particle concentration distribution. The particles were injected from the inside of the valve only. Directed by the high-speed jet flow, the particles emitted through the flap opening are mostly concentrated in the space between the mask (partition wall in the present study) and the high-speed jet, where the recirculation region forms [Figs. 7(a) and 7(b)]. The flow coming out of the valve in the exhalation condition is similar to the offset jet or the flow over the backward-facing step (Eaton and Johnston, 1981; Pelfrey and Liburdy, 1986; Nasr and Lai, 1998; Agelin-Chaab and Tachie, 2011). As the jet flow is ejected, it entrains the surrounding fluid and a region of lower pressure (recirculation region) is formed underneath the jet due to the presence of the solid surface. If such a flow structure is formed closer to the solid surface, the pressure difference across the jet forces the jet to be attracted to the wall, a phenomenon commonly known as the Coanda effect (Reba, 1966; Assoudi *et al.*, 2015). Since this phenomenon is encouraged on a curved surface (Reba, 1966), it is expected that particle trapping under the valve will appear stronger on the actual face mask.

**FIG. 10.**
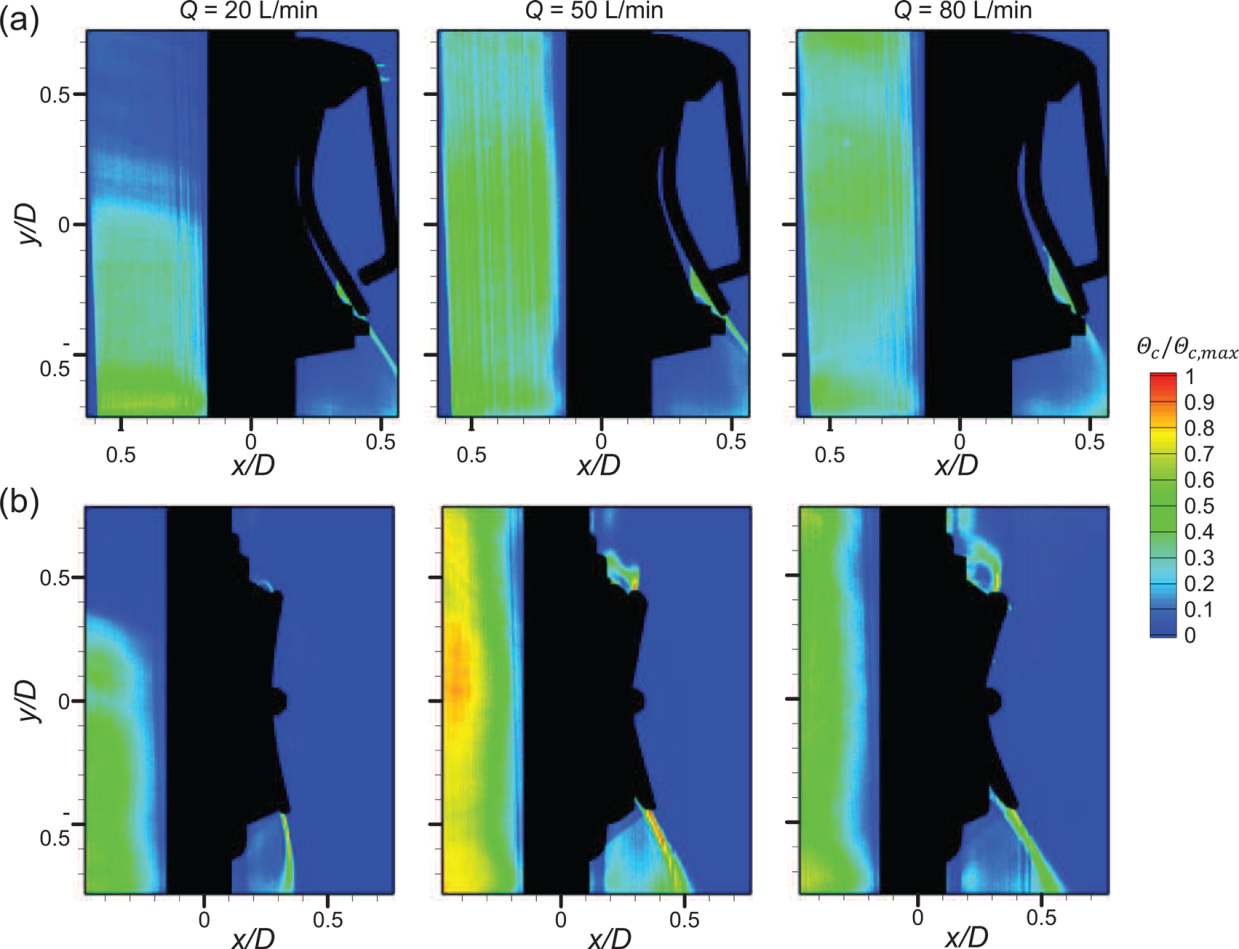
Time-averaged concentration field in the ejection condition: (a) square valve and (b) circular valve.

**FIG. 11.**
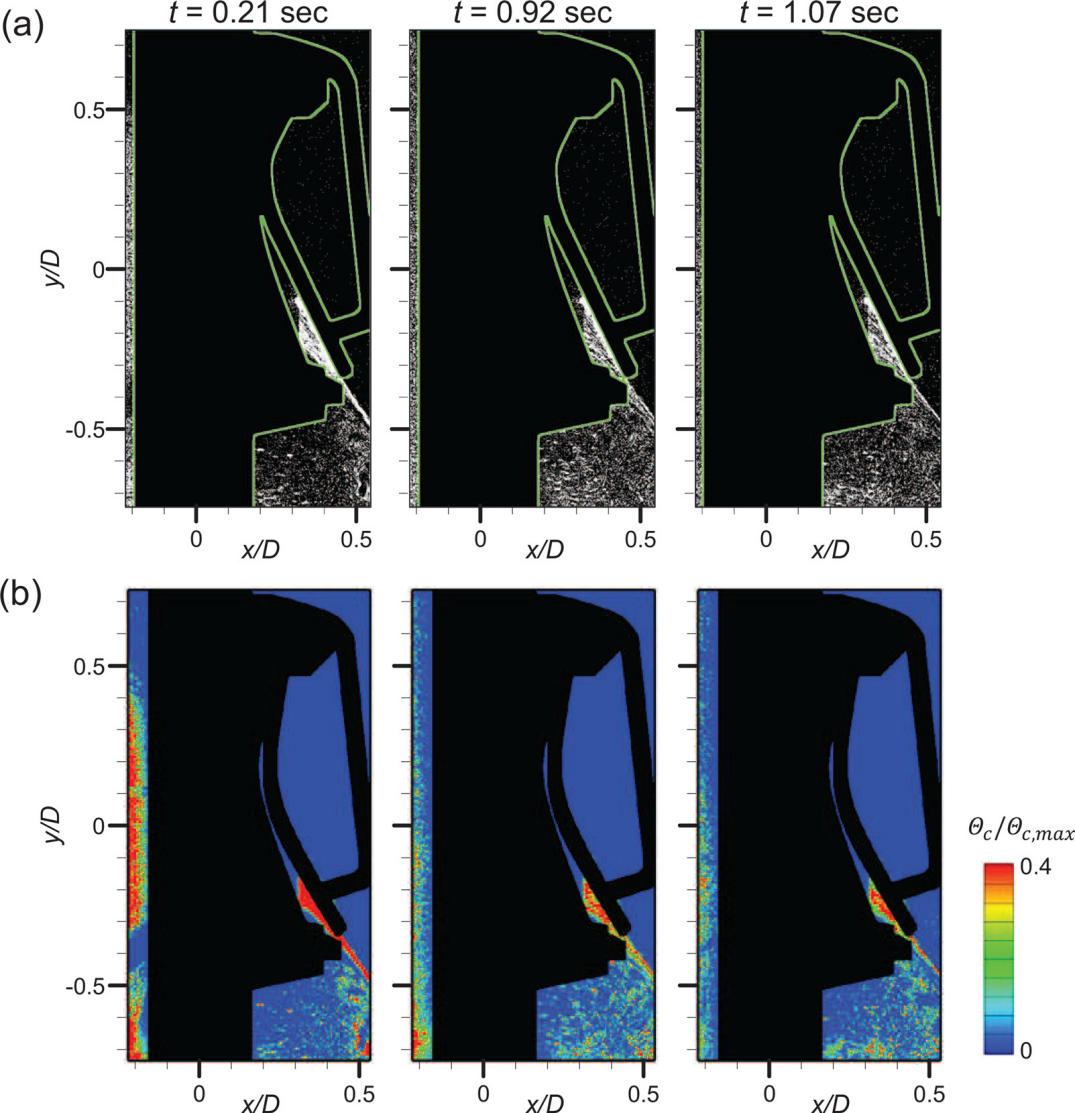
Temporal sequency of particle dispersion in the ejection condition (*Q *=* *50 L/min) for the square valve: (a) particle distribution (raw image) and (b) particle concentration field.

**FIG. 12.**
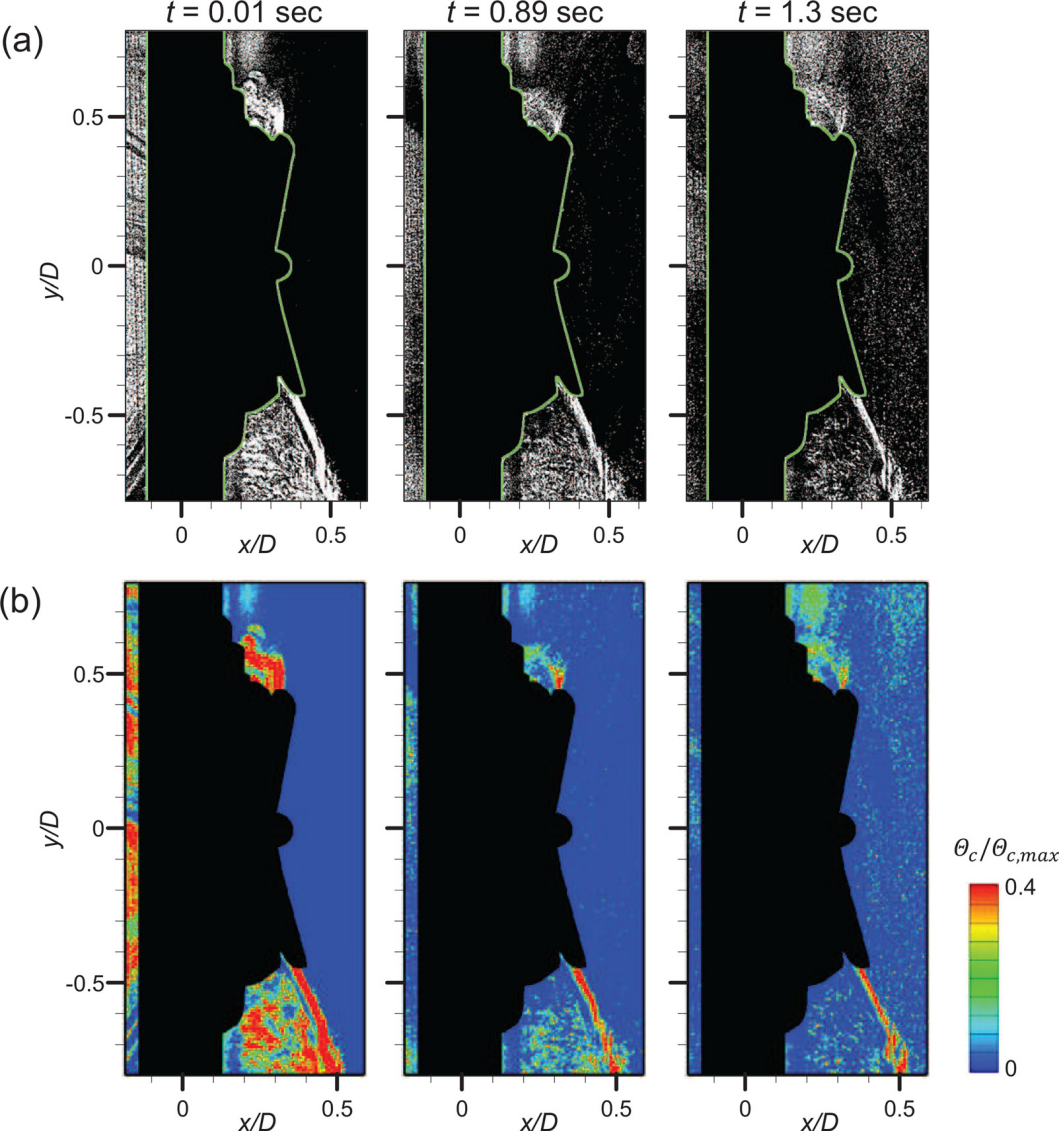
Temporal sequency of particle dispersion in the ejection condition (*Q *=* *50 L/min) for the circular valve: (a) particle distribution (raw image) and (b) particle concentration field.

The concentration of particles is highest along the jet centerline, owing to the high airflow speed and the entrainment of the surrounding air toward the high-speed jet. The particles suspended in the outside region are dragged into the jet, such that there are almost no particles outside the high-speed jet and most of the particles are trapped in the recirculation region [Figs. 9(b) and 9(c)]. The preferential concentration of particles in a time-averaged flow field with varying *Q* is shown in Fig. 10. As shown, the highest particle concentration appears along the high-speed jet for both valve types, and the concentration in the recirculation region appears higher than that of the outer shear layer of the jet. The high-speed jet where the particles are concentrated rolls inward and extends outward for the square and circular valves, respectively, as the airflow rate increases. The pattern of particle spreading over time is different by the valve type, as well. In Fig. 11, for the square valve, particles are trapped and confined in the recirculation region even after time passes. However, for the circular valve, the direction of the particle entrainment is continuously affected by the undulating high-speed jet along the flap circumference. As a result, the particles trapped in the recirculation region at initial time gradually spread outward as time passes (Fig. 12).

### B. Constant suction (inhalation)

**FIG. 13.**
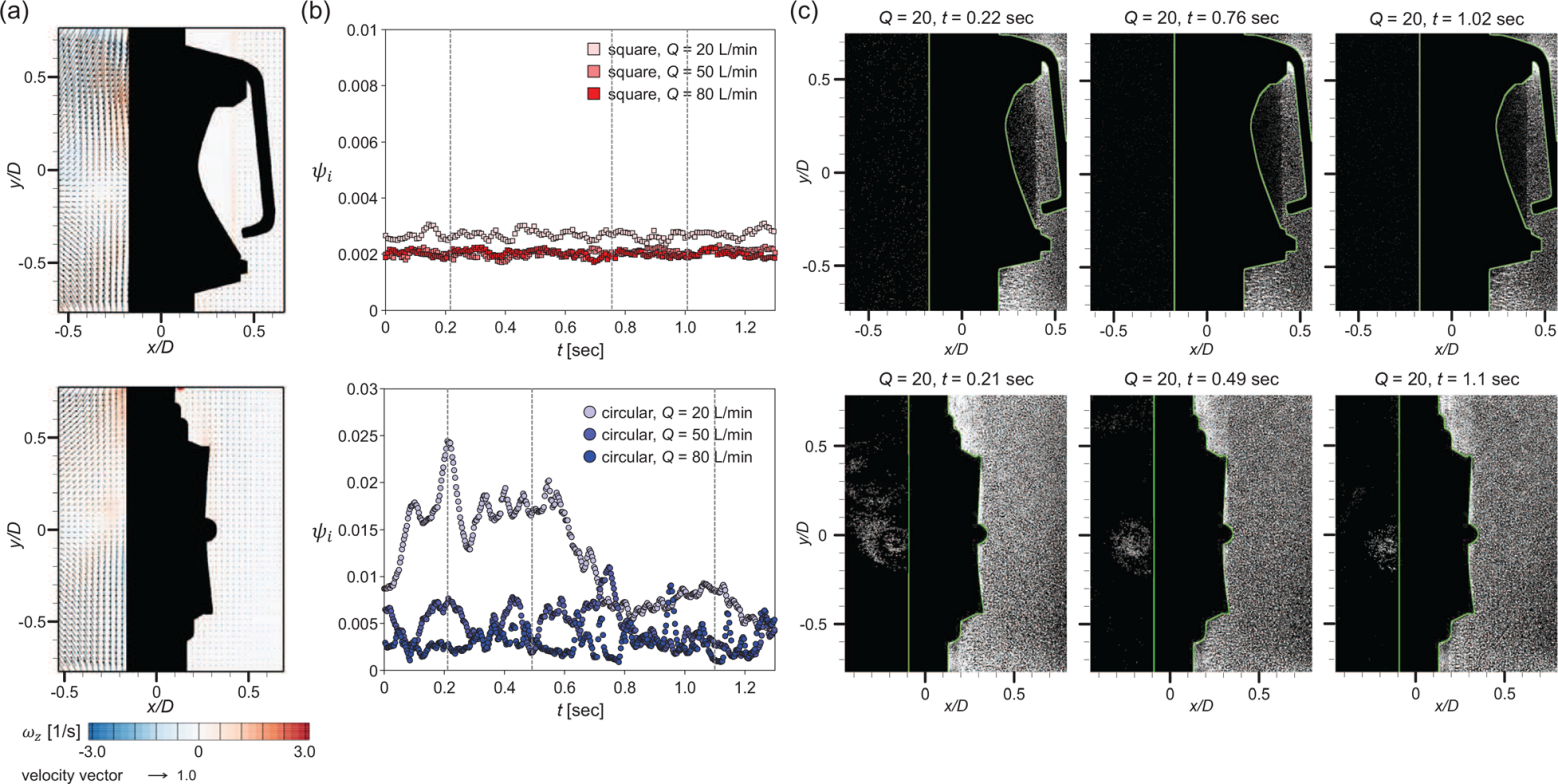
Flow structure and particle dispersion for the constant suction: square (top) and circular (bottom) valves. (a) Time-averaged flow (velocity vectors and vorticity contour) for *Q *=* *50 L/min. (b) Temporal variation of the particle penetration rate toward the valve inside (*ψ_i_*). (c) Particle distribution at the instants highlighted with dashed lines in (b).

To mimic inhalation, we apply suction with a constant flow rate to the valve. When the airflow is suddenly drawn into the valve (corresponding to inhalation), the pressure inside the mask is reduced, which forces the flap to remain closed. As the negative pressure difference across the mask becomes stronger (i.e., with increasing *Q*), the flap adheres more tightly to the valve seat. This is clearly observed in the time-averaged flow fields. Unlike the flow inside the valve, which is dragged in the direction of the suction, the airflow outside the valve is almost quiescent regardless of the valve type [Fig. 13(a)]. Therefore, it is expected that particle penetration, if any, toward the mask inside would occur in the early stage of inhalation.

This can be quantified as a particle penetration rate toward the valve inside (*ψ_i_*), as shown in Fig. 13(b). As in the case of periodic breathing below, the particles were injected from outside the valve. In Fig. 13(b), we compare temporal variation in *ψ_i_* by the valve type. In the inhalation stage, the overall particle penetration rate is not significant (less than 1%), in particular for the square valve [Figs. 13(b) and 13(c)]. More particles are leaked through the circular valve than the square valve, which is most evident at a lower airflow rate (e.g., *Q *=* *20 L/min). This is consistent with Kuo *et al.* (2005), who found that a circular valve type was more susceptible to leakage at lower airflow rates. At *t *<* *0.21 s, *ψ_i_* reaches a peak (up to 2.5%) and then gradually decreases as the suction is maintained [Figs. 13(b) and 13(c)]. This can be elucidated by the characteristics of valve geometry. The circular valve consists of a circular rubber flap lying parallel to the flat valve seat while its central part is affixed. Therefore, the central part of the flap receives the most force from the protruding part of the valve cover, but there is little force (such as tension on the flap) acting on the edge of the flap circumference, resulting in unstable flap holding [see the local particle leakage in Fig. 13(c), which is responsible for the peak of *ψ_i_* in Fig. 13(b)]. However, the seat of the square valve is curved, so the flap always remains bent and is under greater and sustained tension force when closed. Thus, the square valve is superior to the circular valve in terms of preventing particle penetration under constant suction. During the inhalation stage, the air flow may be also leaked through the incomplete face seal (Bellin and Hinds, 1990). According to Perić and Perić (2020), however, the flow locally accelerated along the gap between the face and perimeter of the mask during the inhalation did not change the global flow in the inspiratory direction to the extent such that it affects the operation of the exhalation valve, but only brought a slight decrease in the pressure drop across the mask. Therefore, the results obtained in the present condition are not affected by such flow structures.

### C. Transition flow (exhalation–inhalation)

**FIG. 14.**
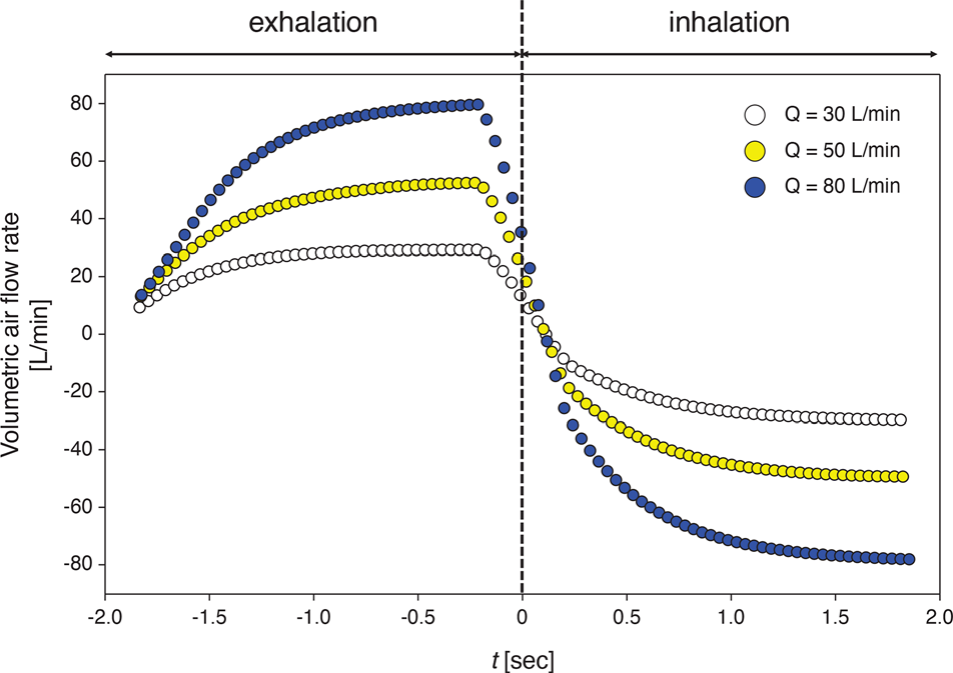
Temporal variation of the volume flow rate of air flow measured for the cyclic flow condition.

In general, in the analyses of constant exhalation and inhalation, the penetration rate during inhalation is not significant, as confirmed by the certification test, which restricts airflow leakage under constant suction; however, substantial numbers of particles are ejected outside during constant exhalation. Thus, in this section, we evaluate the performance of the square and circular valves when the airflow transitions from ejection (exhalation) to suction (inhalation) (i.e., periodic flow condition). Actual human breathing repeats stages of exhalation and inhalation every 5.4 s, while the inhalation is slightly shorter than the exhalation (Gupta *et al.*, 2010). Furthermore, the unsteady effect before the flow reaches the steady condition is shown to be the source of particle penetration through the valve [Figs. 13(b) and 13(c)]. Nevertheless, no study has investigated particle penetration in real time under such a cyclic flow. Here, we measure the airflow fields and accompanying particle migration as the transition from the ejection to suction occurs, in which the duration of each is 2.0 s. Figure 14 shows the temporal variation of the airflow rate measured for each *Q*, which shows a relatively sharp phase transition. Previous studies related to face mask or virus transmission used the modeled human respiratory flow in various ways, mostly based on the results from Gupta *et al.* (2010), such as a sine wave (Khosronejad *et al.*, 2021), partially linear profile (Lei *et al.*, 2013; Zhang *et al.*, 2016; Akagi *et al.*, 2021), and periodic square wave (Dbouk and Drikakis, 2020a). While the detailed profiles may different among each other, the transition rate between exhalation and inhalation is an important factor to determine the unsteady flow structures. For the present cases, the transition rate normalized by the maximum flow rate (*Q*) is approximately 3.0 s^−1^ ( Q=30–80 L/min), which is in the same order of previous studies; it was 1.8 s^−1^ for *Q *=* *24 L/min (Gupta *et al.*, 2010) and 3.1 s^−1^ for *Q *=* *60 L/min (Akagi *et al.*, 2021). While the present transition occurs faster than the normal breathing condition, it is reasonable considering that the phase conversion occurs faster as the respiratory flow rate increases.

**FIG. 15.**
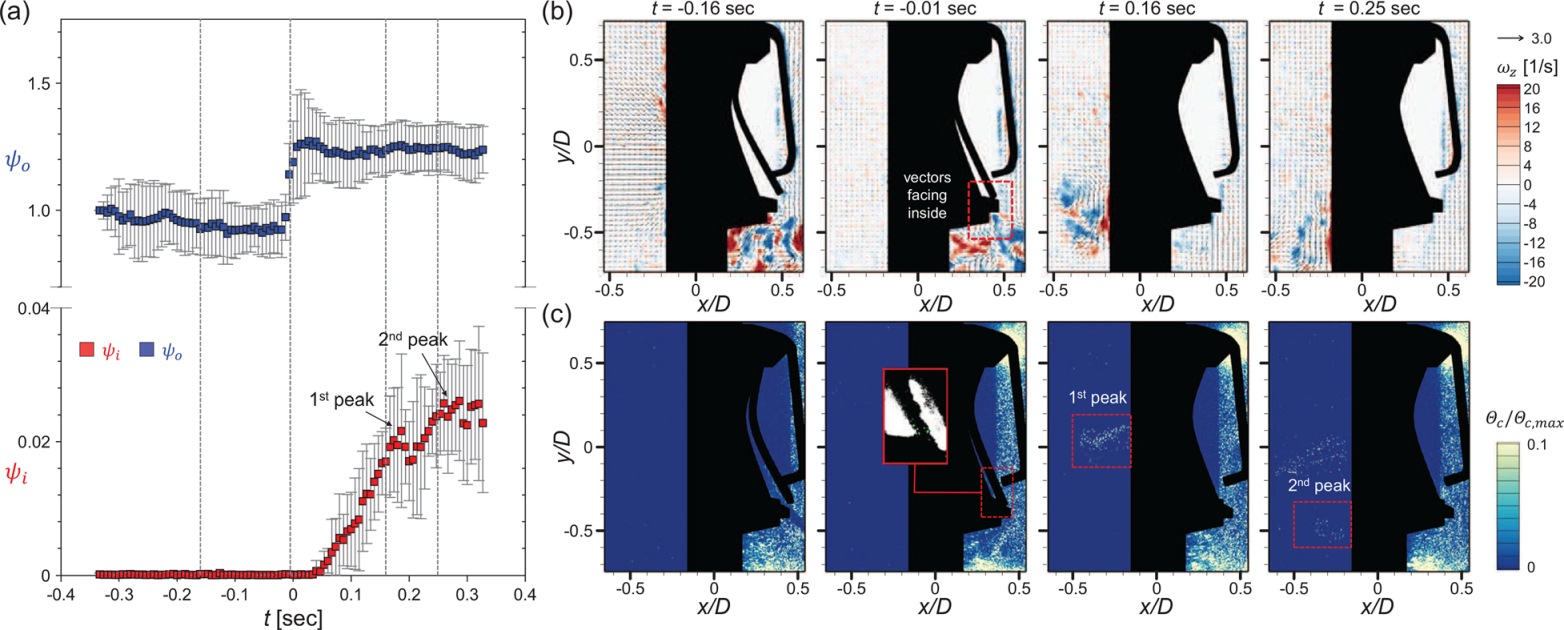
Particle penetration mechanism under periodic breathing for the square valve (*Q *=* *50 L/min). (a) Temporal variation in the particle penetration rates (*ψ_o_* and *ψ_i_*). Airflow field (b) and particle concentration distribution (c) at the instants highlighted with dashed lines in (a).

**FIG. 16.**
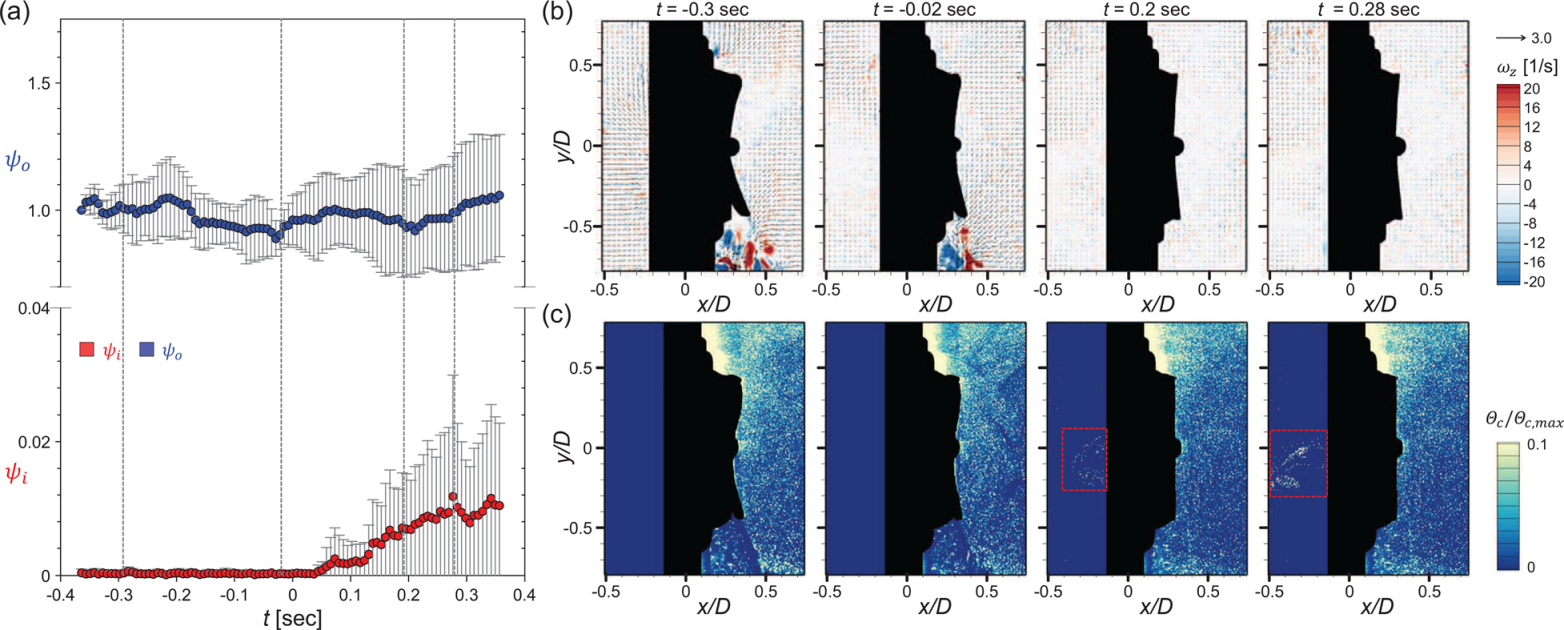
Particle penetration mechanism under periodic breathing for the circular valve (*Q *=* *50 L/min). (a) Temporal variation in the particle penetration rates (*ψ_o_* and *ψ_i_*). Airflow field (b) and particle concentration distribution (c) at the instants highlighted with dashed lines in (a).

The square and circular valves show quite different particle penetration patterns in periodic conditions owing to the differences in the valve structure and induced flow structure. For a representative airflow rate of *Q *=* *50 L/min, temporal variation (the flap closes at *t *=* *0) in the particle penetration rates (*ψ_o_* and *ψ_i_*) is given in Figs. 15(a) and 16(a), respectively, and some representative instants of each phase are investigated further in terms of the instantaneous airflow field [Figs. 15(b) and 16(b)] and particle concentration distribution [Figs. 15(c) and 16(c)]. For the square valve (Fig. 15), at t=−0.16 s (exhalation stage), the airflow develops along the ejection direction continuously. As shown in the time-averaged airflow field [Figs. 7(a) and 7(b)], reverse flow and strong mixing occur below the inner shear layer of the jet, which causes the particles to be preferentially trapped in the recirculation region. In the concentration field, particles outside the mask are pushed out by the high-speed jet so that few particles exist along the high-speed jet centerline. However, surrounding particles are attracted toward the jet owing to the entrainment effect (Fig. 9). In the recirculation zone, the particles are dispersed evenly on account of the vortex structures (which enhance the mixing). Closer to the transition ( t=−0.01 s), the flap gradually closes as airflow in the direction of suction from the jet nozzle begins to be applied. At this time, the velocity vectors facing inward (inside the valve) and clockwise vortices are observed around the narrow gap of the flap, which is still open slightly. Although it is not remarkable in the particle concentration field, the particles penetrate into the valve along the weak suction flow, between the valve seat and the flap, as evidenced in the raw image near the flap [see the inset of Fig. 15(c)]. Since the size of the particles is very small, that is, the particle Stokes number is $O(10^{-4})-O(10^{-3})$, the response time of the particle to the flow appears to be much shorter than that of the rubber flap, which has a larger area. As a result, a considerable number of particles can penetrate into the valve [Fig. 15(a)].

Right after the flap is closed (*t *=* *0.16 s; inhalation), strong vortical structures in the clockwise rotation are formed inside the valve and penetrated particle clusters rise up along them [Fig. 15(c)], which attributes to the first concentration peak of the particle penetration rate (*ψ_i_*) [Fig. 15(a)]. As the suction state persists (*t *=* *0.25 s), additional particle penetration occurs at the bottom of the flap owing to the small trembling (vibration) caused by the instability of the flap. This additionally results in the second peak in *ψ_i_* [Figs. 15(a) and 15(c)]. For the square valve, the particles often penetrate inside as a cluster and follow the flow in the form of a long streak. Meanwhile, the particle penetration rate (outside), *ψ_o_*, also increases as the flap closes in the inhalation stage [Fig. 15(a)] because the particles pushed out and stagnates by the high-speed jet flow in the exhalation stage are still highly concentrated outside the mask even in the inhalation stage.

The results for the circular valve (at the same *Q *=* *50 L/min) are shown in Fig. 16. In the exhalation stage ( t=−0.30 s), the ejection airflow is applied at the valve and the high-speed jet flow is generated in both the up- and downward directions [Fig. 16(b)], along which the particles are the sparsest, similar to the square valve [Fig. 16(c)]. As the transition occurs ( t=−0.02 s), the flap is closing and the high-speed jet is deflected closer to the mask. After the flow switches completely to suction (inhalation; e.g., *t *=* *0.20 and 0.28 s), the particles penetrate into the circular valve persistently in the area near the valve center, as shown in Fig. 16(c). Because the circular valve has a fixed position in the center of the flap, the distance from the fixed position to the end of the flap is only half that of the square valve. Thus, the flap opening area is smaller and the response time of the flap is shorter for the circular valve than the square valve. Nevertheless, owing to the absence of force sealing the flap along the circumferential direction, continuous particle penetration occurs along the edge of the flap, following the flapping motion of the flap [Fig. 16(a)]. On the other hand, the particle penetration (*ψ_o_*) increases less compared to the square valve after the flap closes [Fig. 16(a)]. Since the high-speed jet is formed closer to the surface of the mask [Fig. 7(c)] and the operation radius and opening area of the flap are much smaller for the circular valve, the number of particles pushed out by the high-speed jet during exhalation is smaller. Therefore, the increase in *ψ_o_* during the transition is stronger for the square valve.

**FIG. 17.**
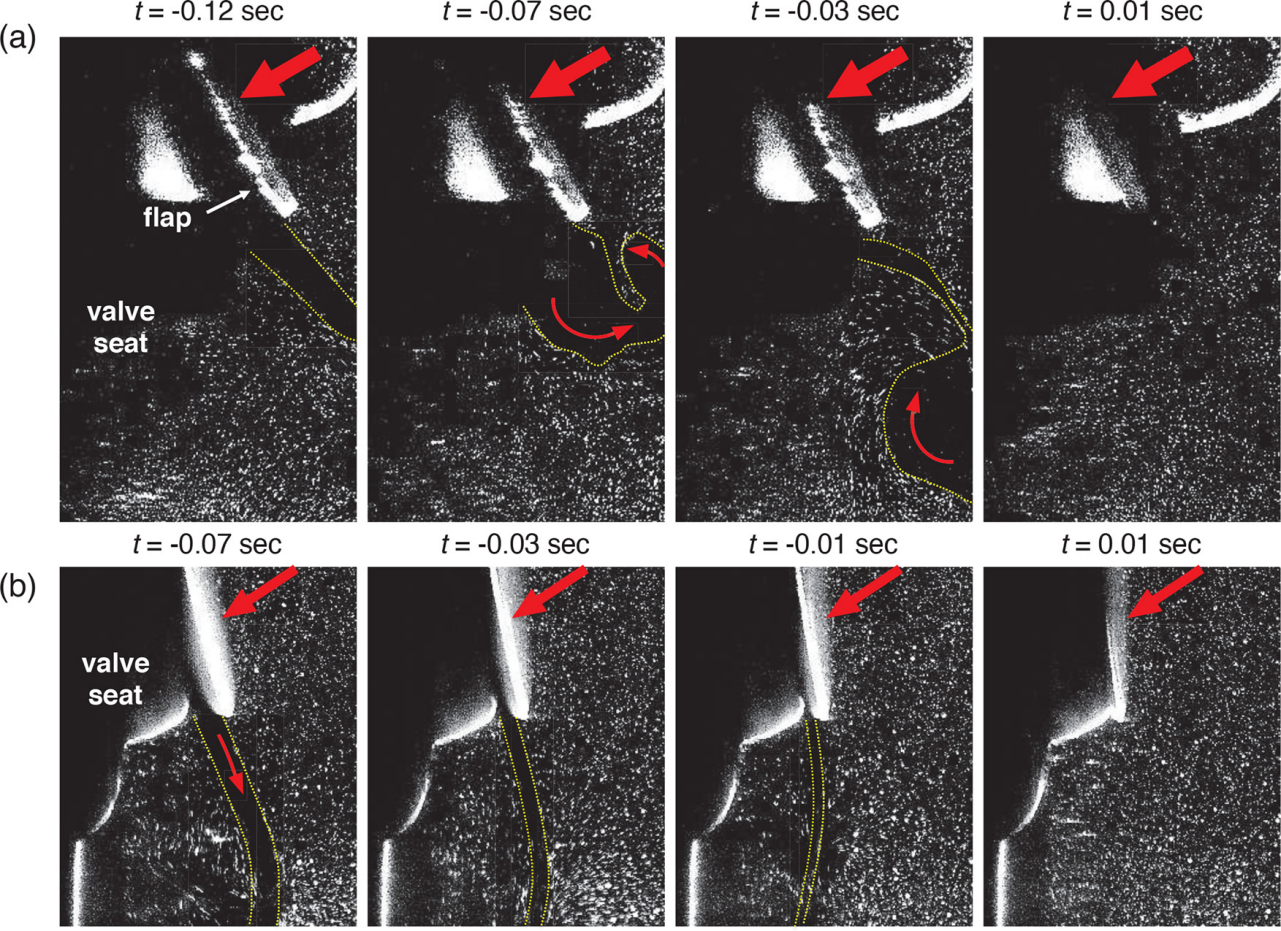
Particle behavior induced by the vortical structures around the jet at the closing moment of the flap in the cyclic flow (*Q *=* *30 L/min): (a) square valve and (b) circular valve.

In Fig. 17, the particle behavior induced by the vortical structures around the fading jet while the flap closes is shown (for *Q *=* *30 L/min), to understand the dispersion pattern of particles induced by the interaction with eddies in the flow. With the square valve [Fig. 17(a)], the particle-laden eddy is formed along the counterclockwise direction owing to the rapid decrease in the ejection flow rate (at t=−0.07 s). As the flow rate decreases sharply, the jet becomes thinner approaching the wall and the clockwise vortex appears leading to the rise of outer particles under the jet (at t=−0.03 s). When the flap closes, the outer particles are quickly dragged into the area of a lower particle population and the uniform distribution is achieved (at *t *=* *0.01 s). For the case of a circular valve, on the other hand, the pattern is different because the thickness of the high-speed jet is thinner and the closing radius is much smaller. As the ejection flow rate decreases, the jet becomes gradually thinner pushing out the outer particles along the jet [Fig. 17(b)], which persists for the entire flow range.

**FIG. 18.**
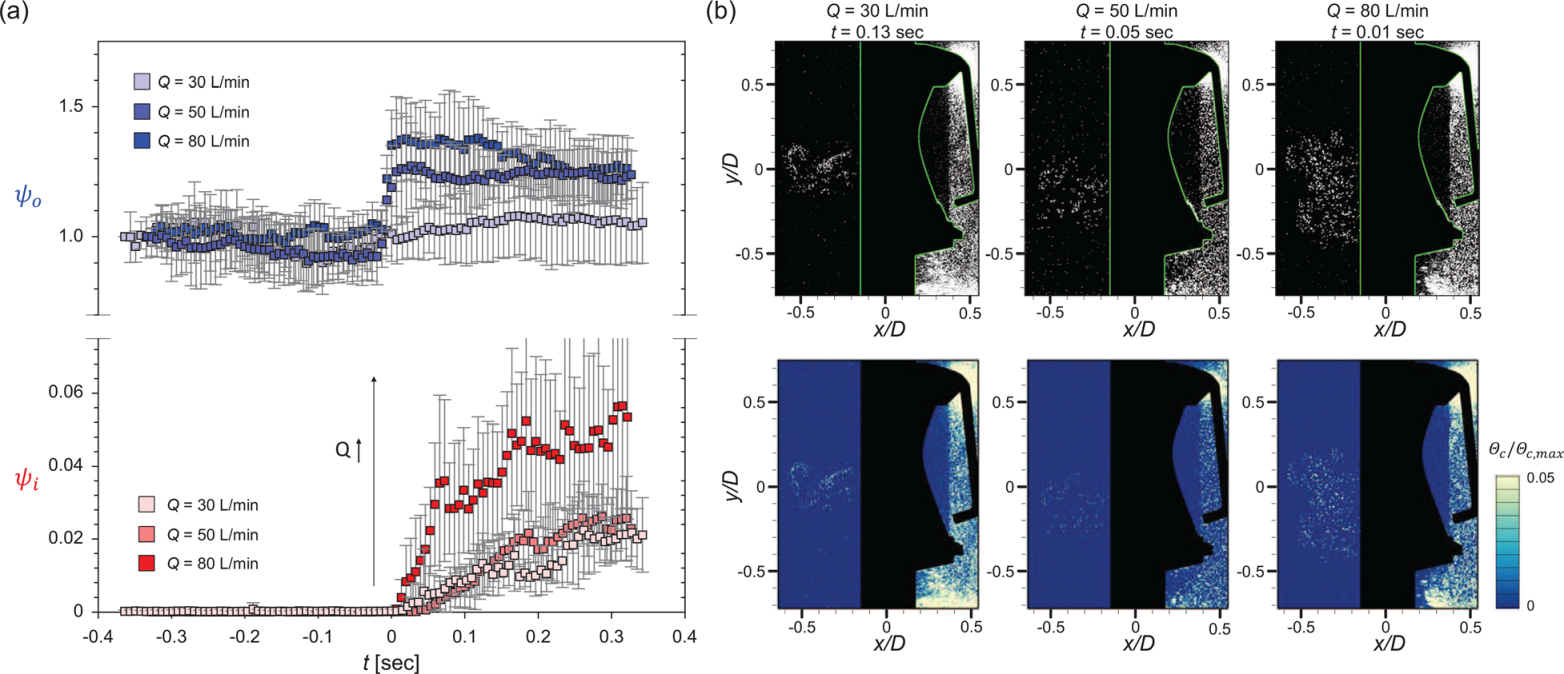
(a) Variation of the particle penetration rate by airflow rate for the square valve under the cyclic flow. (b) Representative instantaneous particle distribution (top) and concentration field (bottom) for each flow rate, after the transition.

**FIG. 19.**
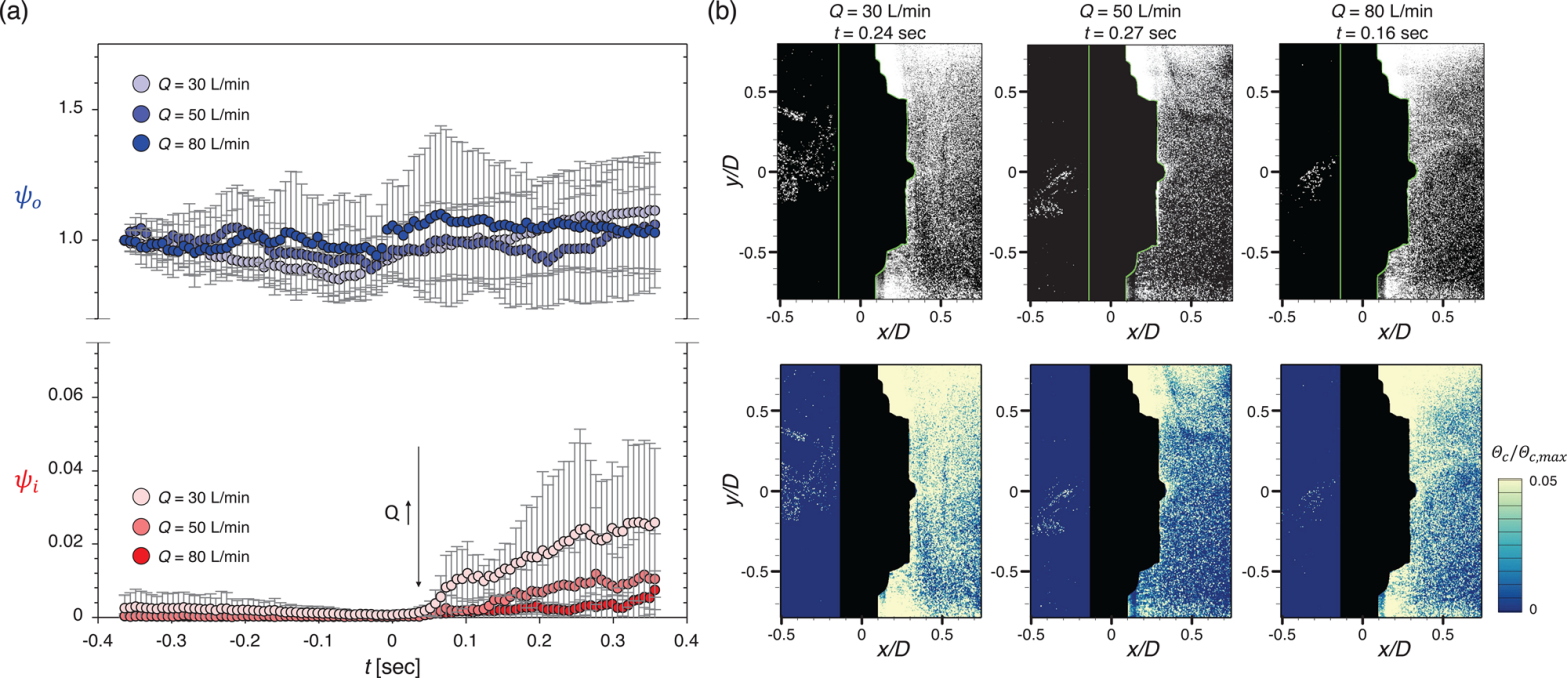
(a) Variation of the particle penetration rate by airflow rate for the circular valve under the cyclic flow. (b) Representative instantaneous particle distribution (top) and concentration field (bottom) for each flow rate, after the transition.

Figures 18 and 19 show the variation in particle penetration rates (*ψ_o_* and *ψ_i_*) in the periodic condition as the airflow rate varies, for the square and circular valve, respectively. It is interesting that the square and circular valves show opposite trends according to the airflow rate (in particular; for *ψ_i_*). For the square valve, particle penetration during the transition increases with increasing *Q* [Fig. 18(a)]. As mentioned above, the major cause of the increase in the penetration rate for the square valve is the instant particle suction that occurs immediately after the flap closes. The flap closes with a large radius and slaps the valve seat, causing instantaneous penetration of particles as a cluster [Fig. 18(b)]. Therefore, a few evident peaks are produced in the particle penetration (*ψ_i_*) profile of the square valve [Fig. 18(a)]. As *Q* increases, penetration increases because the particles are sucked into the flap's opening more quickly; particles penetrating inward are most noticeable when *Q *=* *80 L/min [Fig. 18(b)].

In contrast, the circular valve is more vulnerable to particle penetration at lower *Q* in the periodic airflow condition [Fig. 19(a)]. The main cause of particle penetration through the circular valve is incomplete sealing of the flap (fluttering instability). Since the circular valve has a flapping motion with a relatively small amplitude, few particles penetrate through the open gap as the flap closes. However, the circular valve has a flat valve seat, and there is insufficient force to fix the flap on the valve seat at the flap's edge. Thus, a weak vibration owing to the instability of the flap causes continuous particle penetration along the circumference for the duration of the suction stage [Fig. 19(a)]. As the airflow rate increases, the flap seals the valve seat more tightly, reducing the flap instability and preventing particles from penetrating through the valve. It can be seen that fewer particles penetrate inward as the airflow rate increases [Fig. 19(b)].

This opposite trend in penetration according to the airflow rate in the periodic case is due to the difference in the operation mechanism by the valve type. The length of the flap along the vertical direction for the square and circular valves is 30 and 26 mm, respectively; both valves were designed to be the same scale. However, the radius of the flap movement varies by mechanism; the upper edge of the flap is fixed for the square valve and the central part of the flap is fixed for the circular valve. Thus, under the same ejection flow rate, the flap of the square valve opens wider. This is favorable for lowering the wearer's breathing resistance (internal pressure difference). However, the response time of the flap also increases and significant amount of particle penetration occurs through the gap between the flap and the valve seat while the valve is closed [Fig. 18(a)].

## IV. FURTHER DISCUSSION

### A. The effect of the valve cover

So far, all measurements were conducted with the valve cover removed to clearly visualize the flow (particle movement) across the valve, while some measures were taken to maintain the functioning (kinematics) of the valve. However, in actual commercial masks, the exhalation valve is equipped with a cover on it. To confirm that the present cover-off configuration works the same as an actual cover-on valve, we perform the same flow tests with valves (with cover) for a representative *Q* of 50 L/min. For the breathing type, we considered the same ejection (exhalation), suction (inhalation), and periodic (transition) conditions. Valve covers come in various configurations, usually resembling the shape of the flap and containing gratings to allow the passage of air; the outer shape (typically that of a bluff body) determines the flow around them. For the square valve used in the present study, only the bottom half of the cover includes the gratings. The circular valve, there are relatively dense gratings along the circumference of the cover while the upper part is completely blocked (Fig. 1).

**FIG. 20.**
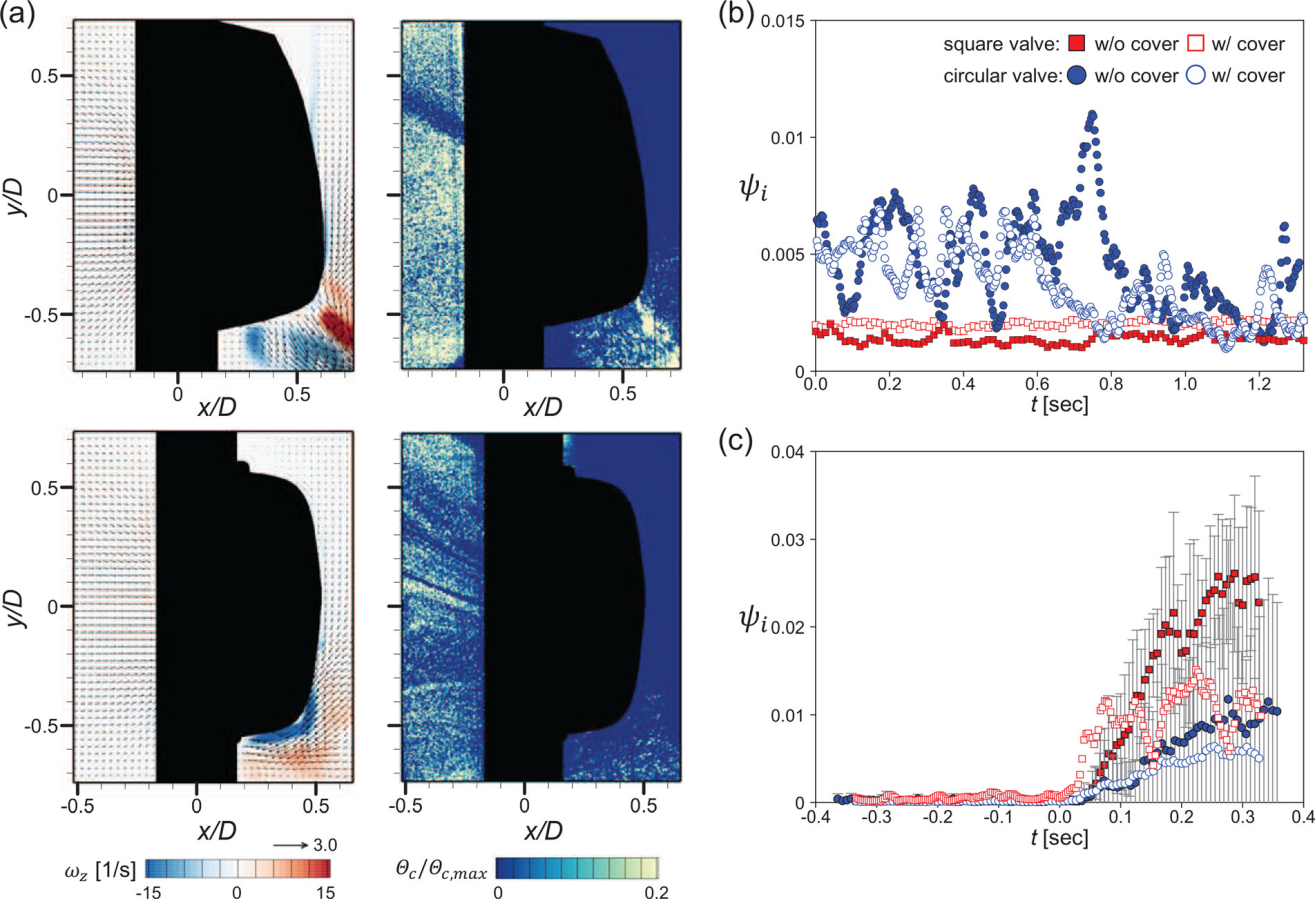
Effect of the valve cover (at *Q *=* *50 L/min) for the square (top) and circular (bottom) valves. (a) Time-averaged flow field (left) and representative instantaneous particle concentration field (right) in the ejection condition. Comparison of penetration rates with and without cover for (b) suction and (c) periodic conditions.

The time-averaged flow field and a snapshot of the representative particle concentration field in the ejection condition for the square and circular valves with covers are shown in Fig. 20(a). For the square valve with cover, the high-speed jet width appears thicker than that of the cover-off valve. This is because the high-speed jet is dissipated through the thin gratings at the bottom of the cover (Fig. 1) with enhanced turbulence. However, the overall characteristics of the flow field—the generation of the high-speed jet below the flap and the entrainment toward this jet centerline—are not altered by the presence of the cover. As can be seen in the instantaneous particle concentration field, the particles inside the valve spread outward rapidly along the high-speed jet [Fig. 20(a)]. Similarly, a recirculation region is formed between the jet and the wall, with particles trapped there. For the circular valve with cover, the top and bottom parts of the cover, where the measurement (center) plane crosses, are blocked. Thus, a slightly different airflow field is generated from that of the cover-off circular valve. Since the high-speed jets are blocked by the cover crossing the center of the valve, this jet is not observed in the time-averaged velocity field. Instead, there are high-speed jets in the off-center planes with a strong entrainment toward the valve (especially at the bottom). Since almost one-third of the upper part of the cover is fully blocked, no particular airflow occurs in the upper part of the circular valve. In the instantaneous concentration field, the particles ejected outside through the high-speed jets are attracted to the bottom of the circular valve, owing to entrainment effect.

Although the airflow and particle distribution across both valves are not affected much by the presence of the cover, we compare the temporal variation in *ψ_i_* for the valves with and without a cover in the suction and periodic conditions, respectively, in Figs. 20(b) and 20(c). With suction (inhalation), there is no significant difference in the particle penetration rate with and without the cover [Fig. 20(b)]. This is because the sealing between the flap and the valve seat, the main cause of the particle leakage, is unaffected by the presence of the cover. In the periodic (exhalation–inhalation transition) case, however, the amount of particle penetration decreases slightly with the cover, but the penetration pattern is similar. This slight decrease is attributed to the presence of empty space between the case and flap and blockage effect by the gratings. Therefore, we believe that the cover-off valve tested in the present study operates almost the same as an actual exhalation valve with a cover.

### B. Estimation of the flow rates through mask filter and valve

Since we have focused on the flow around the valve only, separated from the whole mask, it is meaningful to confirm that the air flow through the valve is dominant. We applied flow the simple resistance circuit model to compare the flow rates through the mask filter and flap opening gap of exhalation valve. The pressure drop across the mask ( ΔP) can be considered to be equal for both path and the flow through each path can be modeled as a parallel circuit. Thus, the airflow rate ratio ( $Q_{G}/Q_{F}$) is inversely proportional to the flow resistance ratio ( $R_{F}/R_{G}$). Here, the subscript *F* and *G* denote the mask filter and opening gap, respectively. For the present geometry, the flow resistance can be obtained as R=L/kA, where *A* is the cross-sectional area of the passage, *L* is the passage length, and *k* is the permeability (viscous resistance coefficient). For the mask filter, typical values are *L *=* *1 mm, *A *=* *180 cm^2^, and $k=8.9\times 10^{9}$ 1/m^2^ (Xi *et al.*, 2020). For the opening gap, we used *L *=* *5 mm, *A *=* *130 mm^2^, and $k=3.5\times 10^{-6}$ 1/m^2^. Here, the permeability of the open gap was estimated using the friction factor of rectangular channel flow; that is, the friction factor $f=76.28/Re_{D_{h}}$ was calculated with a geometric factor of 76.28 for the rectangular cross section (He and Gotts, 2004). Then, the pressure drop through the gap can be obtained, and the permeability is calculated as $k=Q\mu L/A\Delta P=3.5\times 10^{-6}$ 1/m^2^, using Darcy's law. As a result, the ratio of the flow rate is $Q_{G}/Q_{F}=1.76\times 10^{18}$, indicating that the air passage through the gap in the exhalation valve is dominant and that through the mask filter is negligible. Therefore, we think that the flow structure and associated particle dispersion pattern measured in the present study represent the general situation.

## V. CONCLUDING REMARKS

In the present study, we experimentally analyze the operation of square and circular exhalation valves under realistic flow conditions such as ejection, suction, and periodic flow in a wide range of flow rates to mimic human breathing. In each flow configuration, we find solid evidence and mechanisms of particle leakage across the valve, based on the airflow field and particle distribution.

In the ejection (exhalation) phase, the high-speed jet is generated through the narrow opening between the valve seat and flap. The surrounding airflow (and particles) are entrained toward this jet, forming a recirculation region under the jet. As a result of the interaction with this airflow, particles inside the valve quickly spread outward along the high-speed jet and are trapped in the recirculation region. The same spreading pattern would be experienced by the wearer of an actual mask with the exhalation valve as well. The direction of the jet varies depending on the valve type, resulting in different airflow fields and spreading patterns. In the suction (inhalation) phase, when the flap closes, it separates the inside and outside of the valve. However, the source of the particle leakage is the incomplete sealing (instability) of the circumferential edge of the flap (circular valve) especially at lower airflow rate. Overall, the square valve performs better in terms of preventing particle penetration owing to its curved valve seat structure.

For the periodic (transition from exhalation to inhalation) flow condition, the major cause of the particle leakage depends on the valve design. For the square valve, owing to the discrepancy in the response time of particles and flaps to the airflow, particle cluster penetration occurs instant the flap closes. However, for the circular valve, particle penetration occurs steadily over time due to the imperfect sealing of flap, which is attributed to not only the fixed position of the flap but also the shape of the valve seat.

Although exhalation valves in high-filtration rate masks have passed certification procedure (tested only under a certain constant suction head), limitations in their performance are revealed under these flow conditions similar to actual human breathing. Since the same situation as in the periodic condition occurs repeatedly in real human breathing, instantaneous (unsteady) particle penetration in this transient flow condition may compromise the overall filtering efficiency of the face mask. Especially during a pandemic, a mask equipped with an exhalation valve has no effect for the purpose of source control and preventing airborne infectious disease. This type of mask even plays a role of accelerating the spread of the virus through the high-speed jet created from flap opening. Furthermore, in the present study, the airflow and particle distribution around the valve and the location of the recirculation region are described; it was also possible to locate positions in which particles are preferentially concentrated.

In sum, the present study provides useful insights into more rigorous methods of preventing virus spread on disinfection equipment, including face masks. Since the inevitable limitations resulting from the basic structure of an exhalation valve with a flapping motion have been confirmed, it is necessary to develop a better exhalation valve to promote both safety and convenience. Based on the present results, we can expect that a new valve design may compensate for the current limitations of exhalation valves without adding another power source or additional filter. Existing exhalation valves have gratings on the valve cover in the direction the high-speed jet is released (where particle preferential concentration occurs) to allow airflow passage. If the open part (grating) is designed in the center of the cover, for example, the accelerating high-speed jet generated during exhalation will not be able to come out immediately. Instead, the jet would decelerate, circulating inside the cover, and the spread of the internal source would be alleviated. In addition, a simple spiral tooth flow path between the flap and cover, would reduce the instantaneous particle penetration that occurs during cyclic flow by delaying the particle response time.

## Data Availability

The data that support the findings of this study are available within the article.
